# A Functionality‐Graded Cathode Electrolyte Interphase Enables Ultra‐Long Cycling Stability in Aqueous Zn–Mn Batteries

**DOI:** 10.1002/advs.202522338

**Published:** 2026-01-30

**Authors:** Kaisheng Sun, Yanlei Geng, Shengen Gong, Fangfei Li, Liang Li, Xiaoteng Jia, Danming Chao, Caiyun Wang

**Affiliations:** ^1^ Synergetic Extreme Condition High‐Pressure Science Center State Key Laboratory of High Pressure and Superhard Materials College of Physics Jilin University Changchun China; ^2^ College of Chemistry Jilin University Changchun China; ^3^ State Key Laboratory of Integrated Optoelectronics College of Electronic Science and Engineering Jilin University Changchun China; ^4^ Intelligent Polymer Research Institute AIIM Facility, Innovation Campus University of Wollongong North Wollongong NSW Australia

**Keywords:** cathode electrolyte interphase, cyclic stability, electrolyte additives, structure‐function correlation, Zn–Mn batteries

## Abstract

The rational design of cathode electrolyte interphases (CEI) is pivotal for enhancing reaction kinetics and stability in zinc–manganese batteries, yet their design principle and formation mechanisms remain unclear. In this work, we introduce an electrolyte additive‐driven in situ strategy using trace KH_2_PO_4_, guided by theoretical calculations, to construct a functionality‐graded hierarchical CEI on a carbon‐coated Cu‐MnO_2_ cathode. This precisely engineered structure effectively regulates the interfacial water environment, mitigates volume stress, and promotes efficient charge carrier transport. Specifically, the inner amorphous inorganic Zn_3_(PO_4_)_2_/ZnHPO_4_ layer enhances ion transport, the intermediate organic phosphate ester layer with C─O─P bonds provides mechanical flexibility, and the hydrated outer layer with adsorbed $H_{2}{\mathrm{PO}}_{4}^{-}$/${\mathrm{HPO}}_{4}^{2-}$ traps water molecules via hydrogen bonding, suppressing corrosion. As a result, the battery achieves exceptional cycling stability of 100 000 cycles at 5.0 A g^−1^, nearly a 10‐fold improvement over conventional systems. This work presents a universal approach for interfacial engineering in aqueous batteries, offering new insights into regulating CEI formation and reaction kinetics via electrolyte engineering to achieve durable energy storage performance.

## Graphical abstract

Through the interfacial reaction of KH_2_PO_4_, a functionally graded CEI with hydrated, organic, and inorganic layers is constructed. This suppresses interfacial water corrosion, mitigates volume stress, and promotes charge carrier transport, achieving superior battery stability.

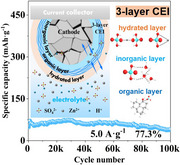

## 1 Introduction

Aqueous Zn–Mn batteries (ZMBs) are promising candidates for large‐scale energy storage devices owing to their abundant resources, safe and low‐cost electrolytes, and high energy density [1, 2, 3, 4, 5, 6]. Their performance, however, is hindered by the intrinsic limitations of manganese‐based cathodes. These include low ionic and electronic conductivity hindering reaction kinetics, and severe lattice stress during ion intercalation/deintercalation processes, leading to structural degradation [7, 8, 9, 10, 11, 12, 13]. Moreover, electrolyte pH fluctuations promote the formation of insulating by‐products [14, 15], while slow Zn^2+^ desolvation increases polarization [16, 17]. To overcome these challenges, researchers have adopted strategies such as electrolyte engineering, structural design, and element doping engineering to improve overall battery performance [18, 19, 20, 21, 22, 23]. The in situ construction of a stable cathode electrolyte interphase (CEI) via electrolyte engineering has proven particularly effective [24, 25], as it improves interfacial conductivity and alleviates volume‐induced stress in manganese‐based materials [26, 27, 28, 29].

Conventional CEI layers are generally categorized into inorganic and organic types [30, 31]. The inorganic CEI, produced by oxidative decomposition of electrolyte anions at high voltages, forms a dense layer with high ionic conductivity and excellent chemical stability that effectively suppresses metal dissolution and electrolyte decomposition [32]. However, it suffers from poor mechanical flexibility and tends to crack under volume changes. In contrast, the organic CEI, formed via oxidative polymerization of organic solvents (e.g., carbonates) or reactions with additives [33, 34], offers greater flexibility to accommodate electrode volume changes but suffers from low ionic conductivity and inferior chemical stability [35, 36]. Organic/inorganic hybrid layered CEIs that integrate the advantages of both types, the mechanical flexibility of organic layers and the chemical stability of inorganic layers, have emerged as a promising alternative. The formation of the CEI is governed by the adsorption and decomposition of ions or solvent molecules. Therefore, introducing trace electrolyte additives has become a key strategy to regulate interfacial chemistry and enhance battery stability by minimizing electrolyte decomposition [37, 38]. Moreover, as the CEI forms on the cathode surface, the interfacial compatibility between the cathode material and electrolyte additives is critical for regulating interfacial properties. For example, the use of fluorocarboxylates, with their strong adsorption and interfacial compatibility with carbonyl materials, forms a CEI that prevents direct contact between the electrolyte and electrode, thereby suppressing dissolution and achieving excellent stability [39]. Despite recent progress, the precise design of functionally graded CEI structures remains challenging due to limited theoretical guidance and inadequate understanding of their formation mechanisms.

Here, an in situ strategy for constructing a functionally graded CEI is developed via electrolyte additive engineering, guided by theoretical calculations, to enhance ZMB performance. Trace KH_2_PO_4_ introduced into the electrolyte regulates interfacial reactions on a carbon‐coated Cu‐MnO cathode. The preferential adsorption and decomposition of $H_{2}{\mathrm{PO}}_{4}^{-}$ anions on C═O sites of the cathode surface drive esterification‐like reactions and self‐decomposition, forming a hierarchical triple‐layer CEI structure with distinct functional characteristics: an inorganic inner layer, an organic intermediate layer, and a hydrated outer layer. The outer hydrated layer consists of $H_{2}{\mathrm{PO}}_{4}^{-}$ and ${\mathrm{HPO}}_{4}^{2-}$ species that immobilize interfacial water molecules via hydrogen bonding, thereby mitigating electrolyte‐induced corrosion. Beneath this layer, an organic intermediate layer, dominated by phosphate ester groups linked by C─O─P covalent bonds, provides flexibility to accommodate volume changes and alleviate mechanical stress. The innermost inorganic layer, consisting mainly of Zn_3_(PO_4_)_2_ and ZnHPO_4_, exhibits high ionic conductivity, facilitating efficient charge transport across the interface. Moreover, the acidic environment provided by KH_2_PO_4_ facilitates the transformation of Cu‐MnO into Cu^2+^‐doped K_x_MnO_2_ (Cu‐MnO_2_) with a high oxidation state and tunnel structure during cycling, while the dissociation of $H_{2}{\mathrm{PO}}_{4}^{-}$ stabilizes the electrolyte pH. The resulting functionally graded CEI significantly enhances battery stability and suppresses manganese dissolution, enabling a cycle life of up to 100 000 cycles at 5.0 A g^−1^, nearly ten times that of conventional systems. This study offers a facile electrolyte‐engineering strategy to tailor cathode interfaces and regulate reaction kinetics, advancing the practical implementation of high‐performance batteries.

## 2 Results and Discussion

### 2.1 CEI Formation Mechanism

**FIGURE 1:**
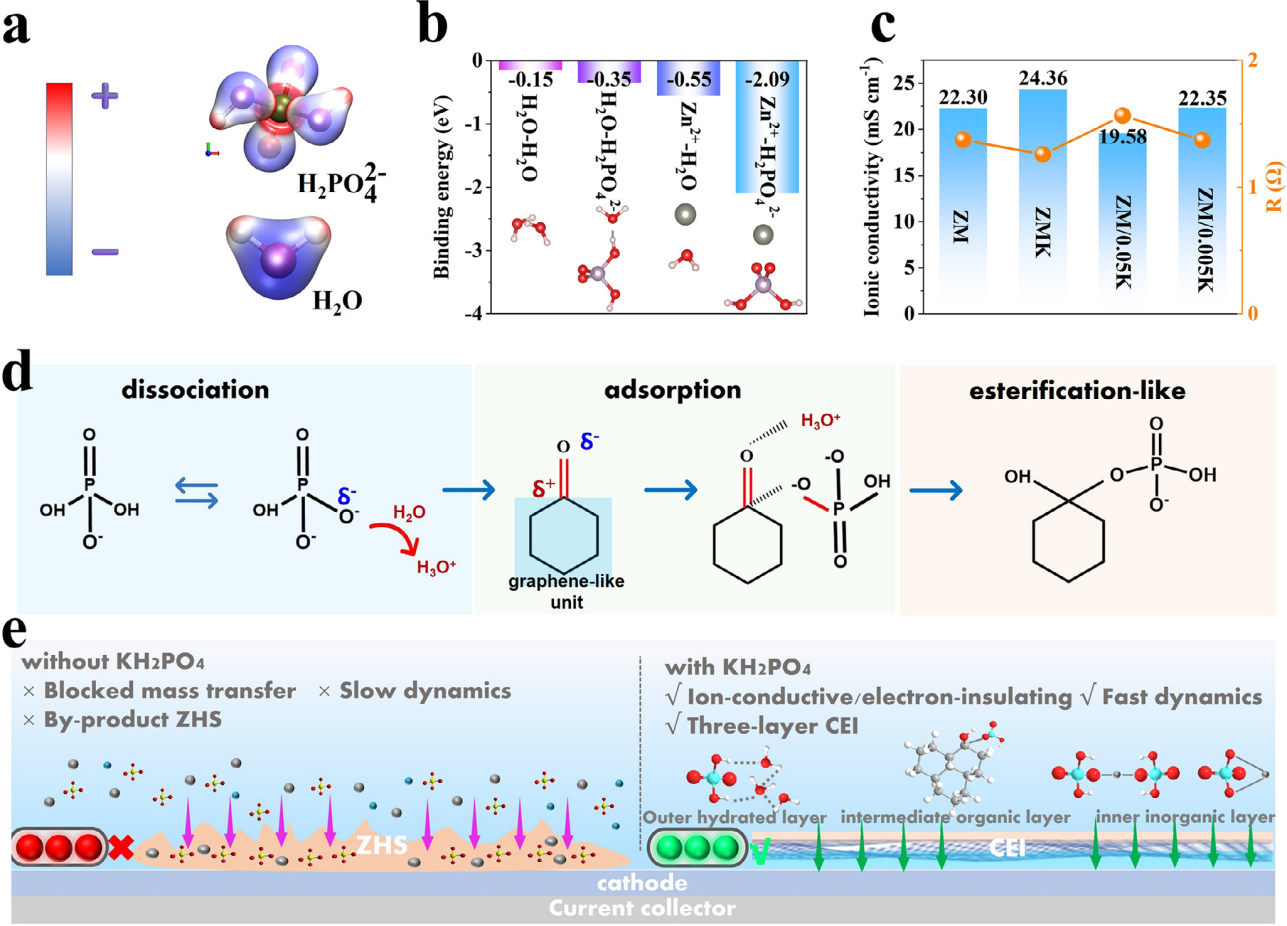
Schematic of the electrolyte additive‐driven design principle for constructing a functionality‐graded CEI. (a) The electrostatic potential mapping of H_2_O and $H_{2}{\mathrm{PO}}_{4}^{-}$. (b) Binding energies of H_2_O─H_2_O, H_2_O‐$H_{2}{\mathrm{PO}}_{4}^{-}$, H_2_O‐Zn^2+^, and Zn^2+^‐$H_{2}{\mathrm{PO}}_{4}^{-}$. (c) Ionic conductivity of KH_2_PO_4_ additives at different concentrations (ZM: 2 m ZnSO_4_ + 0.2 m MnSO_4_; ZMK: Containing 0.02 m KH_2_PO_4_; ZM/0.05K: 0.05 m KH_2_PO_4_; ZM/0.005K: 0.005 m KH_2_PO_4_). (d) The mechanism of in situ formation of CEI. (e) Schematic illustration of the effect of ZM and ZMK electrolytes on the cathode interface.

In AZIB electrolytes, additive molecules significantly influence the hydrogen‐bond network and the Zn^2+^ solvation sheath. The electrostatic distribution was visualized to identify functional sites through electrostatic potential (ESP) mapping. Regions of high electrostatic potential are electrophilicity and tend to accept electrons, while regions of low electrostatic potential and nucleophilicity and tend to donate electrons. For H_2_P $O_{4}^{-}$, the H in ‐OH group shows electrophilicity and can form hydrogen bonds with H_2_O, whereas the O atoms in P─O/P═O bonds are nucleophilicity and can coordinate with Zn^2+^ in the solvation sheath (Figure 1a). To quantify these interactions, binding energy calculations were performed (Figure 1b). The binding energy of H_2_O‐$H_{2}{\mathrm{PO}}_{4}^{-}$ (−0.35 eV) was more negative than that of H_2_O─H_2_O (−0.15 eV), indicating the disruption of original H_2_O─H_2_O hydrogen bonds and the formation of a new, stronger H_2_O─$H_{2}{\mathrm{PO}}_{4}^{-}$ hydrogen bond network, thereby regulating the ion conduction environment and solvation behavior [40, 41]. Similarly, compared to Zn^2+^‐H_2_O (−0.55 eV), the binding energy of Zn^2+^‐$H_{2}{\mathrm{PO}}_{4}^{-}$ (−2.09 eV) was more negative, indicating stronger coordination that reduces the number of coordinated water molecules. Through this regulation of the electrolyte environment, $H_{2}{\mathrm{PO}}_{4}^{-}$ enhances ion conductivity, achieving optimal performance in ZMK (Figure 1c).

The self‐decomposition of additive molecules and their coordination reactions with functional groups on the cathode surface enable the in situ construction of the cathode electrolyte interface (CEI), which plays a crucial role in battery performance. The graphene‐like carbon network surface and abundant carbonyl groups in carboxylate‐based MOF‐derived materials provide active sites that facilitate CEI formation (Figure S1). The adsorption energies calculated on the carbon interface reveal that $H_{2}{\mathrm{PO}}_{4}^{-}$ exhibited a more negative adsorption energy (−1.44 eV) than ${\mathrm{SO}}_{4}^{2-}$ (−0.99 eV) and H_2_O (−0.07 eV) (Figure S2), indicating its strongest adsorption propensity. This preferential adsorption is a key factor for the formation of a stable CEI. In aqueous solution, $H_{2}{\mathrm{PO}}_{4}^{-}$ undergoes a dissociation reaction: $H_{2}{\mathrm{PO}}_{4}^{-}+H_{2}O\;⇄{\;\mathrm{HPO}}_{4}^{2-}+H_{3}O^{+}.$ The O^δ^^−^ of ${\mathrm{HPO}}_{4}^{2-}$ coordinates with the C^δ+^ of the C═O bond in the carbon layer via coordination, forming adsorbed species [C═O···O‐PO_3_H^2−^]. Meanwhile, H_3_O^+^ is attracted to the electron‐rich region of the C═O conjugated system and forms [C═O···H_3_O^+^] through hydrogen bonding, thereby balancing local charge distributions. During charging, the high potential drives further oxidation of ${\mathrm{HPO}}_{4}^{2-}$, which engages in an ester‐like reaction with the electrophilic C^δ+^ of the C═O bond, forming a phosphoester group layer via C─O─P bonding on the carbon surface. Simultaneously, ${\mathrm{HPO}}_{4}^{2-}$ undergoes oxidative decomposition and reacts with Zn^2+^, forming amorphous zinc phosphate or zinc hydrogen phosphate. The adsorbed ${\mathrm{HPO}}_{4}^{2-}$ also binds with free water via hydrogen bonds, forming a hydration layer. Consequently, the CEI evolves into a multilayer composite structure composed of an inner inorganic phosphide layer, a middle C─O─P bond‐rich organic layer, and an outer hydrated buffering layer. Within the hydration layer, active water molecules are stabilized via a hydrogen‐bond network, effectively suppressing corrosion and the formation of Zn_4_SO_4_(OH)_6_·xH_2_O by‐products. The organic layer imparts flexibility to accommodate volume stress and enhances structural stability, while the inorganic layer facilitates ion transport and promotes charge carrier kinetics [42].

### 2.2 CEI Structural Characterization and Kinetic Analysis

**FIGURE 2:**
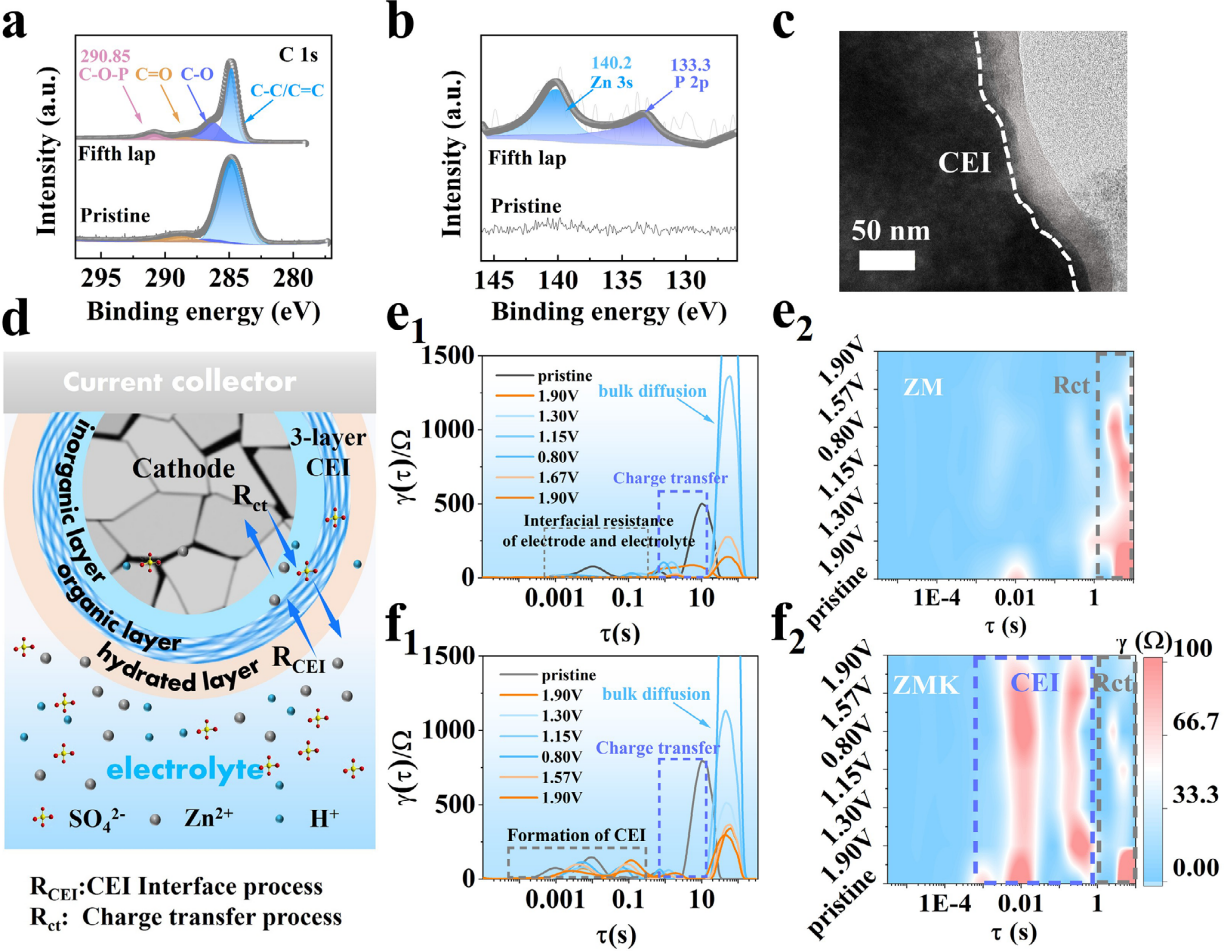
Structural properties of the in situ formed functionally graded CEI. High‐resolution XPS spectra of (a) C 1s, (b) P 2p at pristine and fifth lap. (c) TEM images of Cu‐MnO_2_ after 5 cycles. (d) Impedance distribution of charge carriers during interfacial migration. DRT profiles of the cathode at different voltages in the (e) ZM electrolyte (without KH_2_PO_4_) and (f) ZMK electrolyte (with 0.02 m KH_2_PO_4_).

Interfacial reactions between the cathode and electrolyte under high potentials drive the CEI formation. XRD and XPS analyses of the cathode material after 5 cycles reveal the impact of the KH_2_PO_4_ additive. In the C 1s spectrum (Figure 2a), the intensity of C═O bonds decreased after cycling, while a new peak attributed to C─O─P at 290.85 eV appeared. This indicates that C═O groups act as active sites for the formation and anchoring of the organic layer. In the P 2p spectrum (Figure 2b), the emergence of Zn/P signals after cycling can be ascribed to the mineralization reaction between Zn^2+^ and phosphate groups, confirming the formation of the inorganic layer. In the O 1s spectrum (Figure S3), the significantly increased adsorbed water content supports the proposed mechanism in which KH_2_PO_4_ traps interfacial water via hydrogen bonds to construct a hydrated layer [43].

XRD analysis revealed that MnO transformed into K_x_MnO_2_ after cycling (Figures S4 and S5). In contrast, in the absence of K_x_MnO_2_ (pH≈3.42), MnO was converted to Mn_3_O_4_ [21]. This difference is attributed to the low electrolyte pH (≈3.01) regulated by $H_{2}{\mathrm{PO}}_{4}^{-}$, which facilitates the oxidation of MnO to the +4 state under charged conditions. The Cu 2p spectrum showed that Cu^0^ (932.9 eV) disappeared after cycling and was doped into the K_x_MnO_2_ lattice as oxidized Cu^2+^/Cu^+^ (Figure S6). The surface morphology of Cu‐MnO_2_ after 5 cycles was examined using transmission electron microscopy (TEM). A well‐defined, uniform interface was observed, confirming the formation of a CEI layer. This homogeneously formed CEI layer enhances battery stability by promoting carrier transport and inhibiting electrolyte‐induced corrosion of the electrode.

To probe cathode interfacial kinetics, distribution of relaxation times (DRT) analysis was applied to electrochemical impedance spectroscopy (EIS) to directly decouple overlapping relaxation processes in the time domain, thereby eliminating the subjective bias inherent in equivalent circuit modeling [44, 45]. EIS was measured on Zn//Cu‐MnO_2_ coin cells after 5 pre‐cycles to ensure complete cathode activation and stable CEI formation. DRT analysis deconvoluted the interfacial kinetics into CEI‐related (R_CEI_) and charge transfer (R_ct_) processes (Figure 2d). The DRT curves at various potentials in ZM and ZMK electrolytes (Figure 2e,f) showed peaks in the 10^−4^–10^0^ s range corresponding to R_CEI_, while peaks in the 10^0^–10^1^ s range were associated with R_ct_ [39]. Compared with ZM, ZMK exhibited higher polarization resistance in the R_CEI_ region due to its in‐situ formed electronically insulating yet ion‐conductive CEI that limits electrode–electrolyte contact and suppresses electrolyte decomposition. In the R_ct_ region, both electrolytes showed increased resistance during discharge and decreased resistance during charge; however, ZMK exhibited significantly lower R_ct_, facilitated by its ion‐conductive CEI and large‐tunnel Cu‐MnO_2_ structure. In contrast, Zn_4_(OH)_6_SO_4_·xH_2_O byproducts formation in ZM restricted carrier transport, leading to increased R_ct_. The diffusion coefficients of the ZM/ZMK system were compared using the Galvanostatic Intermittent Titration Technique (GITT), which further confirmed that CEI‐encapsulated Cu‐MnO_2_ exhibited outstanding ion transport capabilities (Figures S7 and S8).

### 2.3 Electrochemical Performances

**FIGURE 3:**
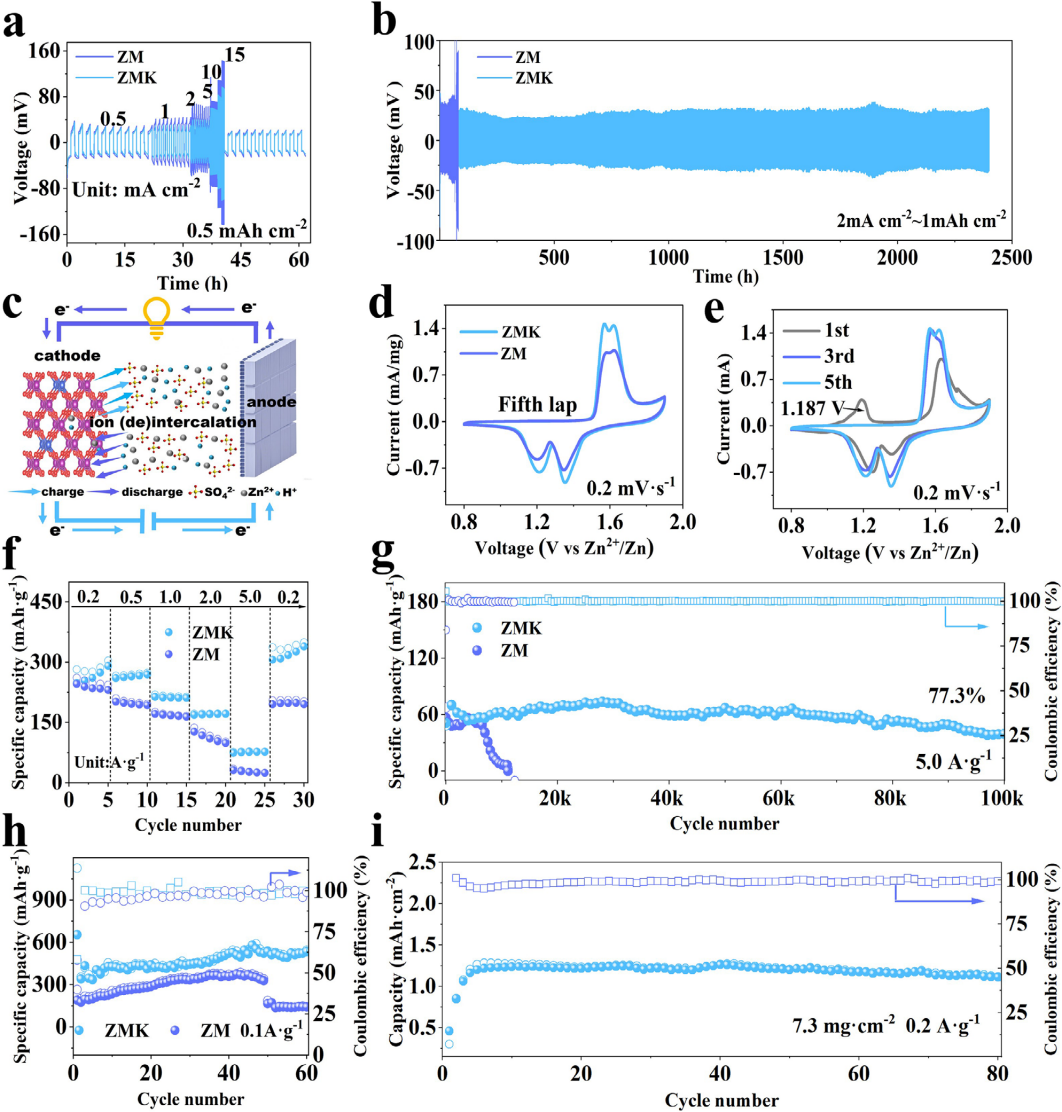
Electrochemical performance of Symmetrical and full batteries in ZM and ZMK electrolytes. (a) Rate performances of symmetrical batteries in ZM and ZMK electrolytes. (b) The cycle performance of Zn//Zn symmetrical batteries in ZM and ZMK electrolytes. (c) Full battery structure sketch. (d) *C*–*V* curves of full batteries in ZM and ZMK electrolytes at the fifth cycle. (e) *C*–*V* curves of full batteries in the ZMK electrolyte at 0.2 mV s^−1^. (f) Rate performance and (g) Long cycle test at 5.0 A g^−1^ (The 21st cycle, after battery stabilization, was selected as the first cycle after activation). (h) Cycling performance at 0.1 A g^−1^ in ZM and ZMK electrolytes. (i) Cycling stability under high load conditions.

Battery stability was evaluated using both symmetric and full‐cell configurations. The ZMK electrolyte in a symmetrical battery exhibited significantly reduced overpotential, especially at high current densities (Figure 3a), and demonstrated exceptional cycling stability, maintaining stable operation for over 2400 h at 2 mA cm^−2^ and 1 mAh cm^−2^ (Figure 3b). This improved performance can be attributed to the in situ formation of a solid electrolyte interface (SEI) layer derived from the decomposition of KH_2_PO_4_ on the Zn anode surface, which enhances interfacial stability and kinetics. The SEI formation originates from a local pH increase induced by the HER, which drives the conversion of adsorbed phosphate species into insoluble Zn_3_(PO_4_)_2_·xH_2_O. Cross‐sectional EDS analysis after 10 cycles (Figure S9) showed a Zn‐, P‐, and O‐enriched interface, providing direct evidenced of the formation of a phosphate‐based SEI on the Zn surface. The presence of phosphorus signals in the XPS P 2p spectra (Figure S10), along with notably reduced impedance and characteristic electric double‐layer peaks (10^−5^–10^−3^ s) observed in DRT analysis (Figure S11), further confirms the formation of this protective SEI [46].

Figure 3c illustrates the full battery structure and its working principle, where Zn^2+^/H^+^ reversibly intercalate/deintercalate into the positive electrode. Synergistic optimization of the anode, electrolyte, and cathode yielded a full battery with enhanced electrochemical activity (Figure 3d). The irreversible oxidation peak observed at 1.187 V in the first‐cycle CV curve (Figure 3e) can be attributed assigned to Cu oxidation, which is consistent with the valence state changes detected in the Cu 2p XPS spectra (Figure S6). These results collectively confirm the successful doping of oxidized Cu into the host material. During galvanostatic charge‐discharge tests, the ZMK electrolyte system demonstrated superior rate capability (Figure 3f). Remarkably, at 5 A g^−1^, it achieved exceptional long‐term cycling stability, retaining 77.3% capacity after 100 000 cycles (Figure 3g), significantly outperforming most reported cathodes (Table S1). At a low current density of 0.1 A g^−1^ (Figure 3h), the electrode still maintained excellent cycling stability, further confirming the stability of both the ZMK electrolyte system and the constructed interface. Moreover, even under a high mass‐loading of 7.3 mg cm^−2^, the system delivered good electrochemical performance, highlighting its strong potential for practical application (Figure 3i).

### 2.4 Battery Stability Analysis

**FIGURE 4:**
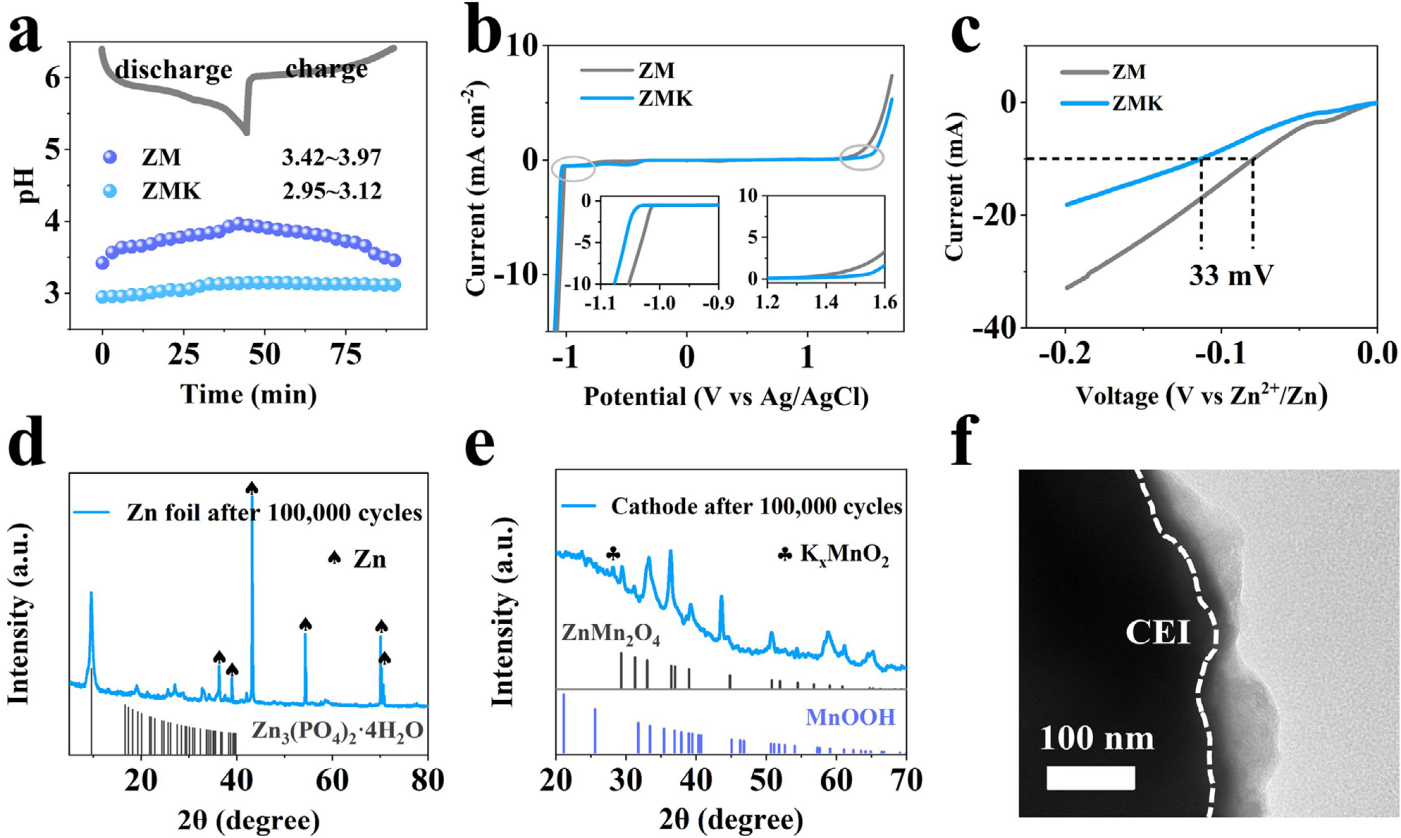
Evaluation of battery cycling stability. (a) In situ pH monitoring at different voltages. (b) Electrochemical stability window of the ZM and ZMK electrolytes. (c) LSV of the ZM and ZMK electrolytes. XRD patterns of (d) anode and (e) cathode after 100 000 cycles. (f) TEM image of the cathode after 100 000 cycles.

Battery health depends on the stability of the electrolyte, anode, and cathode. In situ pH monitoring during cycling in a two‐electrode beaker cell revealed significant fluctuations (3.42–3.97) for the ZM electrolyte, whereas the ZMK remained stable (2.95–3.12) (Figures 4a; S12). This stability stems from KH_2_PO_4_’s buffering effect via the reversible reaction of $H_{2}{\mathrm{PO}}_{4}^{-}$↔H^+^ + ${\mathrm{HPO}}_{4}^{2-}$, balancing pH during proton intercalation/deintercalation. XRD spectra of zinc foils after 7 days of immersion reveal that the KH_2_PO_4_ additive facilitates the formation of Zn_3_(PO_4_)_2_·4H_2_O (PDF#77‐1297) on the zinc anode surface, effectively suppressing the generation of zinc hydroxysulfate (Figure S13). The electrochemical stability window (ESW) of the ZMK electrolyte was significantly broadened due to the suppressed H_2_O activity (i.e., HER and OER) by $H_{2}{\mathrm{PO}}_{4}^{-}$/${\mathrm{HPO}}_{4}^{2-}$(Figure 4b). Due to the physical barrier effect of the SEI, the accessibility of active sites to H_3_O^+^ is reduced, thereby significantly suppressing HER. This is confirmed by the lower hydrogen evolution overpotential in the linear sweep voltammetry (LSV, Figure 4c). Tafel analysis (Figure S14) confirms the superior corrosion resistance of the cell in ZMK electrolyte, as evidenced by the significantly elevated corrosion potential and reduced corrosion current density, consistent with prior reports [47, 48].

Post‐10 000‐cycle electrode analysis reveals the origin of the exceptional stability. XRD analysis indicated that a Zn_3_(PO_4_)_2_·4H_2_O‐based SEI layer formed on the anode surface, effectively suppressing the generation of zinc hydroxysulfate byproduct (Figure 4d). SEM images revealed a uniform and compact SEI layer (Figure S15), in contrast to the large zinc dendrites observed in the ZM electrolyte (Figure S16). These results demonstrate that the constructed SEI layer prompts uniform zinc deposition and suppresses dendrite growth. At the fully discharged state, the XRD pattern (Figure 4e) showed that the cathode retained the well‐defined crystallinity of ZnMn_2_O_4_ and MnOOH. TEM images showed that the initially uniform and dense CEI layer evolved into a non‐uniform structure with a thickness of 24–63 nm after prolonged cycling, yet remained continuous and fully coated the cathode particles without delamination or fracture (Figure 4f). These results collectively demonstrate the robustness of the cathode structure, which withstands repeated lattice changes and volume stress induced by ion intercalation and deintercalation.

### 2.5 Theoretical Calculations

**FIGURE 5:**
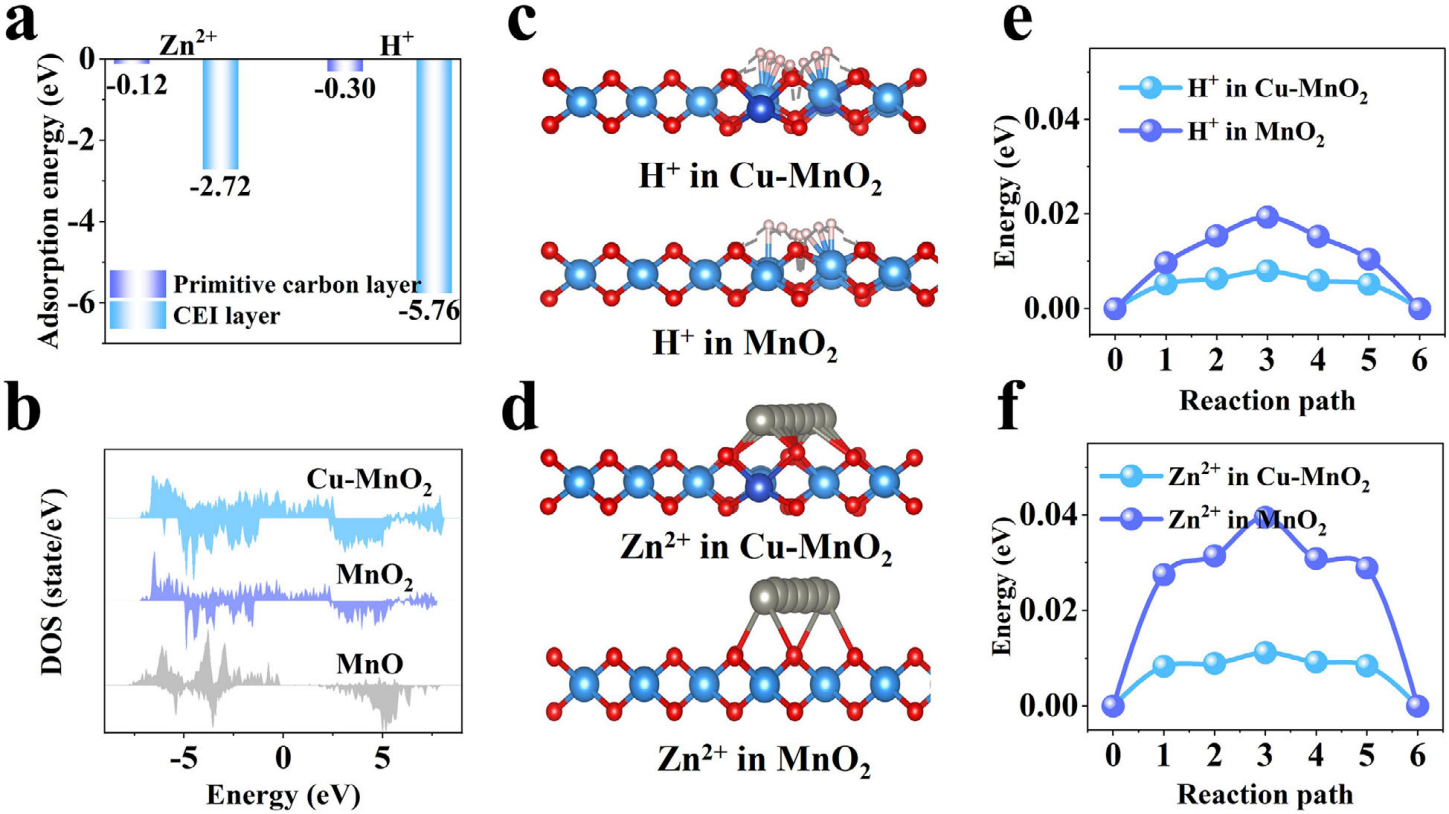
DFT analysis of cathode material kinetics. (a) Adsorption energies of Zn^2+^/H^+^ on the primary carbon layer and CEI layer. (b) DOS files of MnO, MnO_2_ (no Cu^2+^ doping), and Cu‐MnO_2_. (c,d) H^+^/Zn^2+^ migration pathways in Cu‐MnO_2_ and MnO_2_. (e,f) H^+^/Zn^2+^ migration energy barriers in Cu‐MnO_2_ and MnO_2_.

To elucidate the mechanism underlying the excellent performance of the Cu‐MnO_2_ composite, density functional theory (DFT) calculations were carried out. Figure 5a compares the adsorption energies of Zn^2+^/H^+^ on the pristine carbon layer and the KH_2_PO_4_‐induced CEI layer. The results reveal that the CEI layer exhibits a significantly enhanced adsorption of Zn^2+^/H^+^, which facilitates their capture from the electrolyte, promoting carrier transport across the interface. Furthermore, the density of states (DOS) was employed to investigate the electronic conductivity of the manganese oxide component within the CEI layer (Figure 5b). Compared to the pristine MnO, both Cu‐doped MnO_2_ and undoped MnO_2_ showed a continuous DOS near the Fermi level, indicating favorable electronic conductivity. Moreover, Cu‐MnO_2_ showed a higher density of states near the Fermi level, suggesting more free electrons available for charge transport and highlighting the favorable influence of Cu doping on the electronic structure. Comparison of the migration energy barriers for H^+^/Zn^2+^ in Cu‐MnO_2_ and MnO_2_ (Figure 5c–f) reveals that Cu‐MnO_2_ exhibits the lowest barrier, indicative of superior ion transport capability. These findings indicate that the Cu‐MnO_2_ composite features an externally ion‐conductive yet electrically insulating CEI and an internally mixed ionic/electronically conductive crystal. This functionally hierarchical structure enhances interfacial ion transport, reaction kinetics, and structural stability, thereby leading to improved overall battery performance.

## 3 Conclusion

In summary, this work presents an effective electrolyte additive strategy to overcome the intrinsic ionic/electronic transport limitations and structural instability of manganese‐based materials, significantly enhancing ZMB performance. A functionally graded CEI, consisting of an inner inorganic layer, a middle organic layer, and an outer hydrated layer, is successfully constructed on carbon‐coated Cu‐MnO_2_ cathodes using trace KH_2_PO_4_, effectively regulating the interfacial aqueous environment, alleviating volumetric stress, and promoting efficient carrier transport. Guided by theoretical calculations, this CEI design effectively regulates the interfacial aqueous environment, alleviates volumetric stress, and promotes efficient carrier transport. DFT simulations further revealed that the CEI significantly enhances the adsorption energy of carriers, while the electrolyte‐induced tunnel‐type Cu‐MnO_2_ improves electronic conductivity and lowers the ion diffusion energy barrier. The resulting ZMB achieves an ultra‐long cycling life of 100 000 cycles at 5.0 A g^−1^, nearly an order of magnitude improvement over the conventional systems. This work highlights the critical role of rationally designed hybrid CEI layers in stabilizing Mn‐based cathodes and regulating interfacial behavior. It provides fundamental insights into structure‐performance relationships and presents a practical electrolyte engineering approach for developing durable, high‐performance aqueous zinc–manganese batteries.

## Funding

The National Natural Science Foundation of China (22275066), and the Jilin Provincial Science and Technology Department (20250102123JC), and the Synergetic Extreme Condition User Facility (SECUF).

## Conflicts of Interest

The authors declare no conflicts of interest.

## Supporting information




**Supporting File**: advs74058‐sup‐0001‐SuppMat.docx

## Data Availability

The data that support the findings of this study are available from the corresponding author upon reasonable request.
