# Supplementary material for: A Functionality‐Graded Cathode Electrolyte Interphase Enables Ultra‐Long Cycling Stability in Aqueous Zn–Mn Batteries
Source: Adv Sci (Weinh). 2026 Jan 30;13(19):e22338. doi: 10.1002/advs.202522338 (PMC13045384; doi:10.1002/advs.202522338)
Supplement: Supplementary file 1 — Supporting File: advs74058‐sup‐0001‐SuppMat.docx [file ADVS-13-e22338-s001.docx]

Supporting Information

A Functionality-Graded Cathode Electrolyte Interphase Enables Ultra-Long Cycling Stability in Aqueous Zn-Mn Batteries

Kaisheng Sun, Yanlei Geng, Shengen Gong, Fangfei Li, Liang Li,* Xiaoteng Jia,* Danming Chao,* Caiyun Wang*

K. Sun, Y. Geng, F. Li, L. Li,

Synergetic Extreme Condition High-Pressure Science Center, State Key Laboratory of High Pressure and Superhard Materials, College of Physics, Jilin University, Changchun, 130012, China

E-mail: lliang@jlu.edu.cn

K. Sun, S. Gong, D. Chao,
College of Chemistry, Jilin University, Changchun, 130012, China

E-mail: chaodanming@jlu.edu.cn

X. Jia,

State Key Laboratory of Integrated Optoelectronics, College of Electronic Science and Engineering, Jilin University, Changchun, 130012, China

E-mail: xtjia@jlu.edu.cn

C. Wang,

Intelligent Polymer Research Institute, AIIM Facility, Innovation Campus, University of Wollongong, North Wollongong, NSW 2500, Australia

E-mail: caiyun@uow.edu.au

**Experimental section**

***Precursor MOF synthesis:***

Mn(CH_3_COO)_2_⋅4H_2_O (2.499 mmol), Cu(CH_3_COO)_2_ (0.3409 mmol), and polyvinyl pyrrolidone (PVP, 150 mg) were dissolved in a mixed solvent of ethanol/H_2_O (50 mL/50 mL) under stirring to form Solution A. Separately, 1,3,5-benzenetricarboxylic acid (H_3_BTC, 5.353 mmol) was dissolved in another ethanol/H_2_O mixture (50 mL/50 mL) under stirring to form Solution B. Solution B was then slowly added to Solution A under stirring for 1 h. The mixture was allowed to stand at room temperature for 24 h. The resulting precipitate was collected by washing, dried under vacuum at 60 °C for 12 h, yielding CuMn-BTC.

***Preparation of Cu-MnO:***

Cu-MnO was obtained by calcining precursor MOF, under N_2_ at 700 °C for 2 h (heating rate: 5 °C/min).

***Electrode preparation and battery assembly:***

Electrochemical evaluation used CR2032 coin cells. The cathode slurry contained active material, Ketjen Black, and PVDF (mass ratio 7:2:1). DMF was added dropwise to the powder mixture under stirring to form a uniform slurry. The slurry was coated onto either a stainless-steel wire mesh (mass loading ~ 1.0 mg cm^-2^). Coated electrodes were dried at 80 °C for 12 h under vacuum.

The button cell consists of a zinc foil anode, a cathode, a glass-fiber separator, and an electrolyte. The electrolyte is a solution containing 2 M ZnSO_4_ and 0.2 M MnSO_4_, with the addition of 0.005 M, 0.02 M, or 0.05 M KH_2_PO_4_.

***Electrochemical measurements:***

Cyclic Voltammetry (CV): Conducted on a CHI660E electrochemical workstation (China) within a voltage window of 0.8-1.9 V at scan rates ranging from 0.2 mV s^-1^. Electrochemical Impedance Spectroscopy (EIS): Measured at open circuit voltage using a frequency range of 0.01 Hz to 10,000 Hz and an AC amplitude of 5 mV. The distribution of relaxation times (DRT) was calculated from the EIS data using the open-source MATLAB-based software DRT Tools. Galvanostatic Charge-Discharge (GCD): Performed on a Neware battery testing system (China) between 0.8 V and 1.9 V. The electrochemical stability window (ESW) of the electrolyte was measured by linear voltammetry using Pt sheets as the working and counter electrodes, and an Ag/AgCl reference electrode, at a scan rate of 2 mV/s. The potential was scanned cathodically from the open-circuit potential (OCP) to -1.1 V and anodically to 1.6 V.

***Materials characterizations:***

X-ray diffraction (XRD): Measured on an X-ray powder diffractometer (Cu Kα radiation, λ = 0.15406 nm) to analyze the crystal structure. X-ray photoelectron spectroscopy (XPS): Conducted on a Thermo ESCALAB 250 XI system using monochromatic Al Kα radiation to determine the elemental compositions and valence states. Scanning electron microscopy (SEM): Performed on a LEO1430VP instrument (Germany) to examine the morphology of materials' nanostructures. Transmission electron microscopy (TEM): Carried out on an FEI Tecnai G2 F20 S-Twin microscope (America) operating at 200 kV to investigate the nanostructure morphology and elemental mapping/distribution.

***Supplementary Methods:***

The diffusion coefficients of ions were measured by GITT method. The diffusion coefficient was as follows:

$$\text{D=}\frac{\text{4}}{\text{πτ}}{\text{(}\frac{\text{m}_{\text{B}}\text{V}_{\text{M}}}{\text{M}_{\text{B}}\text{S}}\text{)}}^{\text{2}}{\text{(}\frac{\text{∆}\text{E}_{\text{s}}}{\text{∆}\text{E}_{\text{τ}}}\text{)}}^{\text{2}}$$

Where τ was the constant current pulse time, the $\text{m}_{\text{B}}$and $\text{M}_{\text{B}}$ was the mass and molecular mass of MnO. The S was the electrode-electrolyte interface area. The $\text{∆}\text{E}_{\text{s}}$ was the steady-state voltage change caused by the current pulse. The $\text{∆}\text{E}_{\text{τ}}$ were voltage changes during the constant current pulse.

Two pieces of stainless steel were used as symmetrical electrodes for ionic conductivity measurements, one piece of glass fiber as a separator, and 70 μL of electrolyte in the CR2032 cell. The ionic conductivity of the electrolytes was calculated as:

$$\text{δ}\text{ }\text{=}\frac{\text{L}}{\text{S R}}$$

Where L is the distance between the 2 pieces of stainless steel, S is the area of the piece of stainless steel, and R is the resistance value (EIS) obtained from EIS.

***Theoretical Calculations:***

First-principles calculations were performed using the Vienna ab initio simulation package (VASP) based on density functional theory (DFT). The general gradient approximation (GGA) with the Perdew-Burke-Ernzerhof (PBE) formulation for exchange-correlation functions was employed in this study. A convergence criterion of 1.0 × 10^-5^ V for energy was set for geometry optimization, and all forces on each atom were required to be smaller than 0.02 eV Å^-1^. The cutoff energy for the plane-wave basis set was established at 500 eV. A sampling of the Brillouin zone utilized a Monkhorst-Pack gamma-centered grid of special k points with a 3 × 3 × 1 configuration.


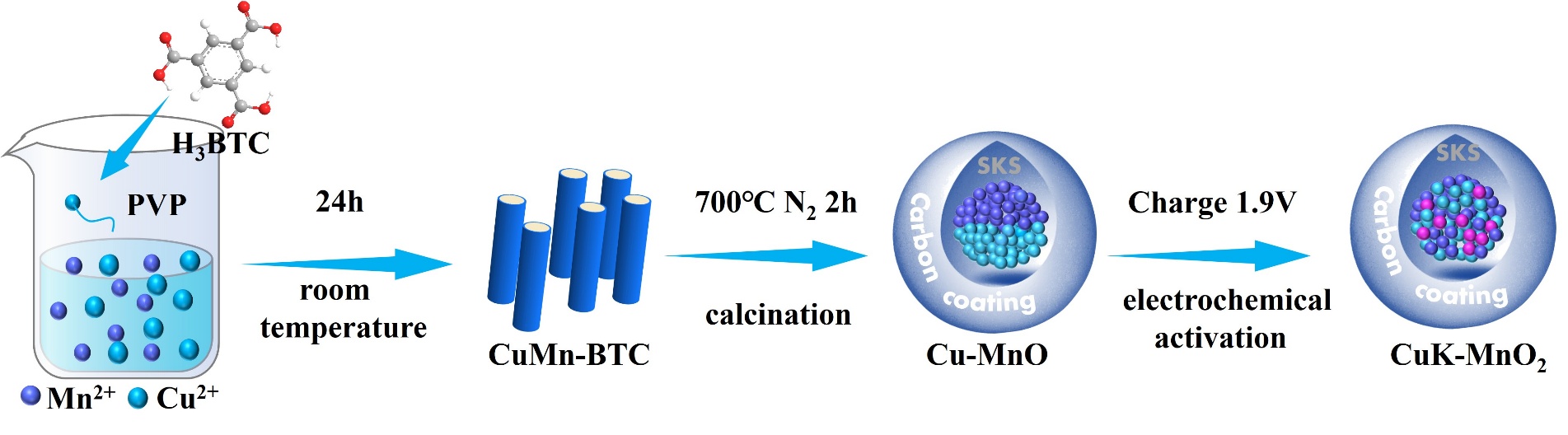


**Fig. S1.** Schematic illustration of the formation of Cu-MnO_2_.

Fig. S1 schematically illustrates the cathode synthesis and electro-activation process. A bimetallic MOF (CuMn-BTC) was first synthesized via coordination between acetate salt (Cu(CH_3_COO)_2_, Mn(CH_3_COO)_2_⋅4H_2_O) and 1,3,5-Benzenetricarboxylicacid. Calcination of this precursor under N_2_ atmosphere yielded a Cu-MnO heterostructure with carbon-coated structures.


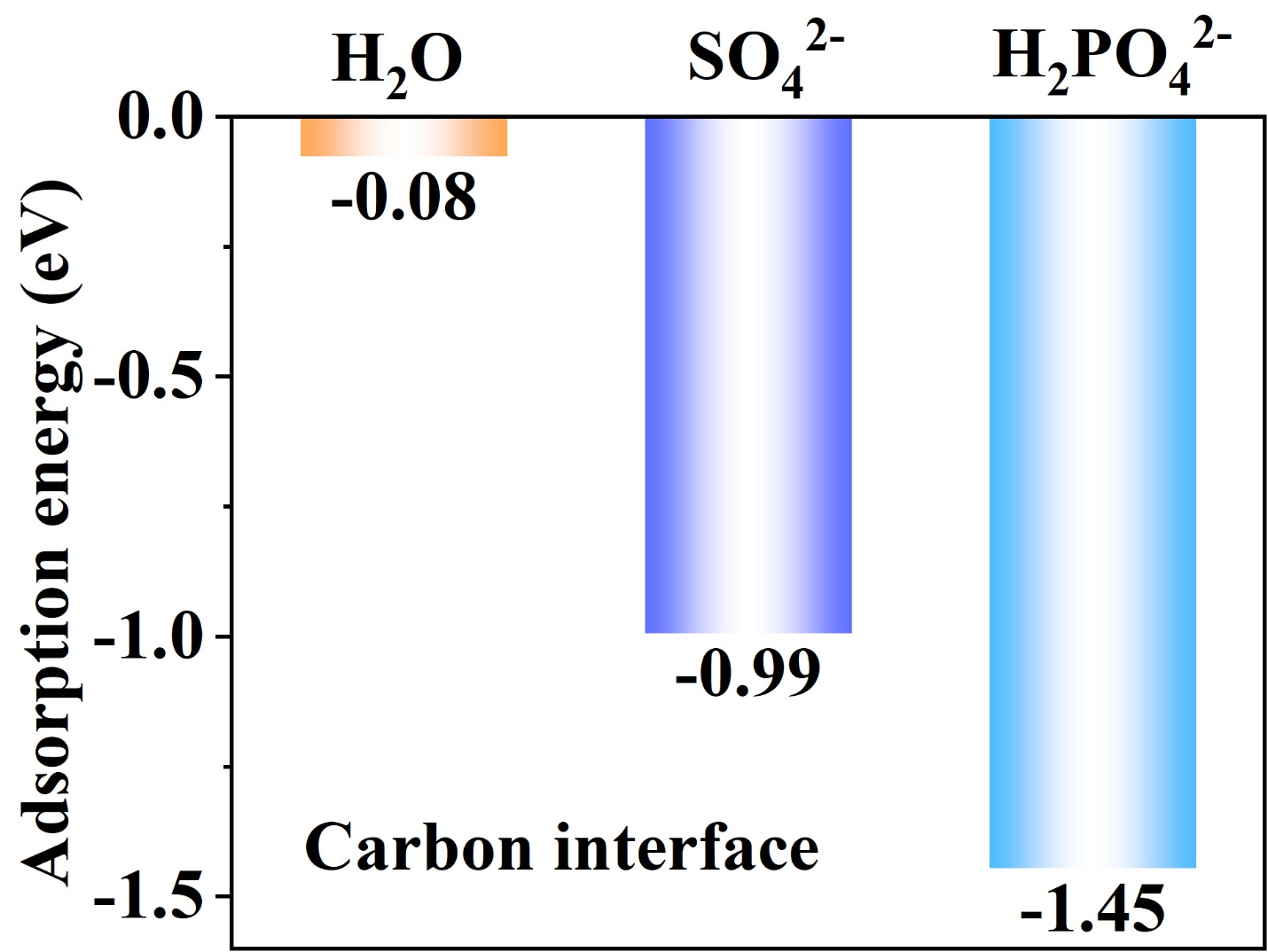


**Fig. S2.** Adsorption energies of H_2_O/$\text{S}\text{O}_{\text{4}}^{\text{2}\text{-}}$/$\text{H}_{\text{2}}\text{P}\text{O}_{\text{4}}^{\text{-}}$ on the primary carbon layer.


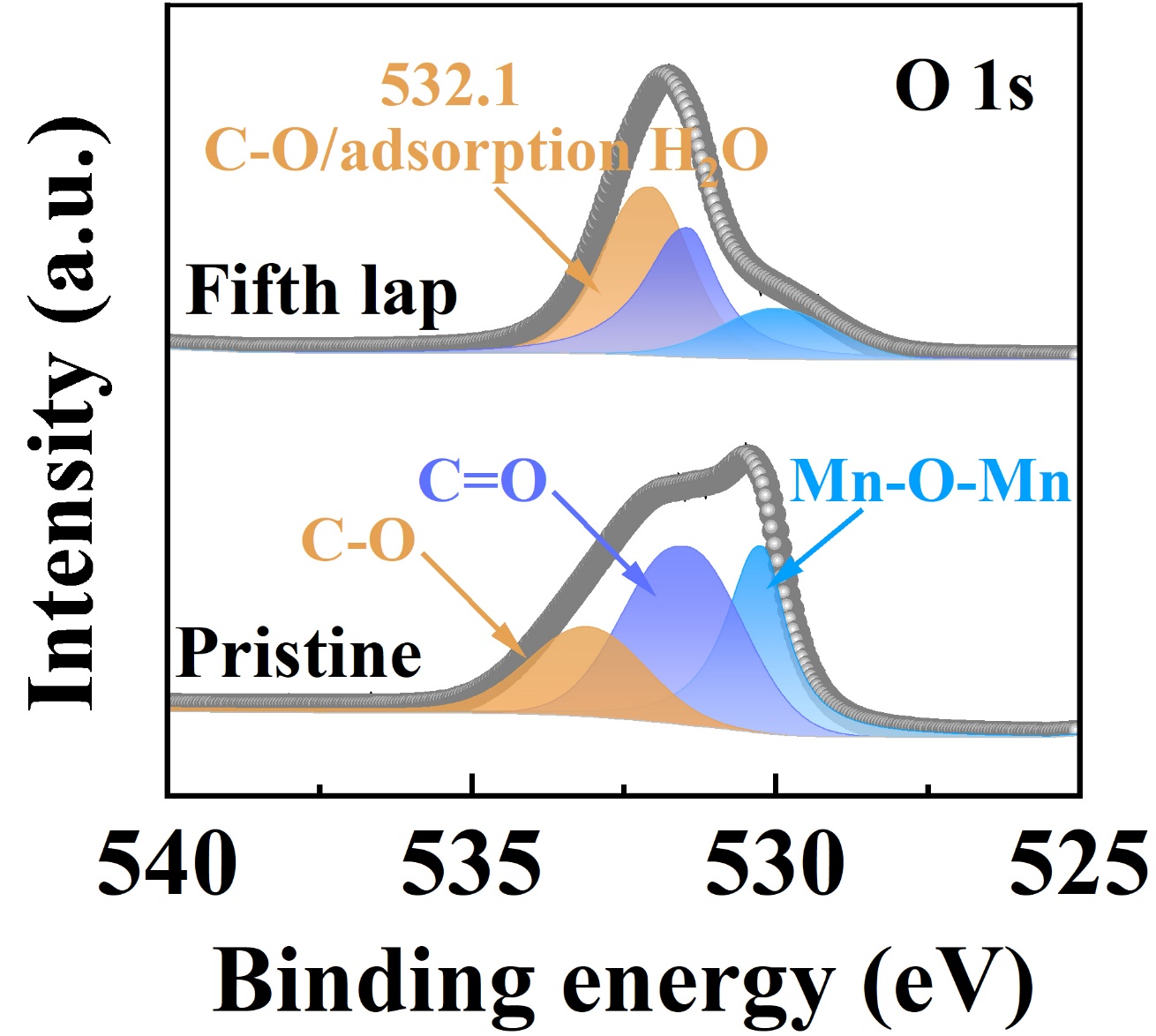


**Fig. S3**. High-resolution XPS spectra of O 1s at pristine and fifth lap.


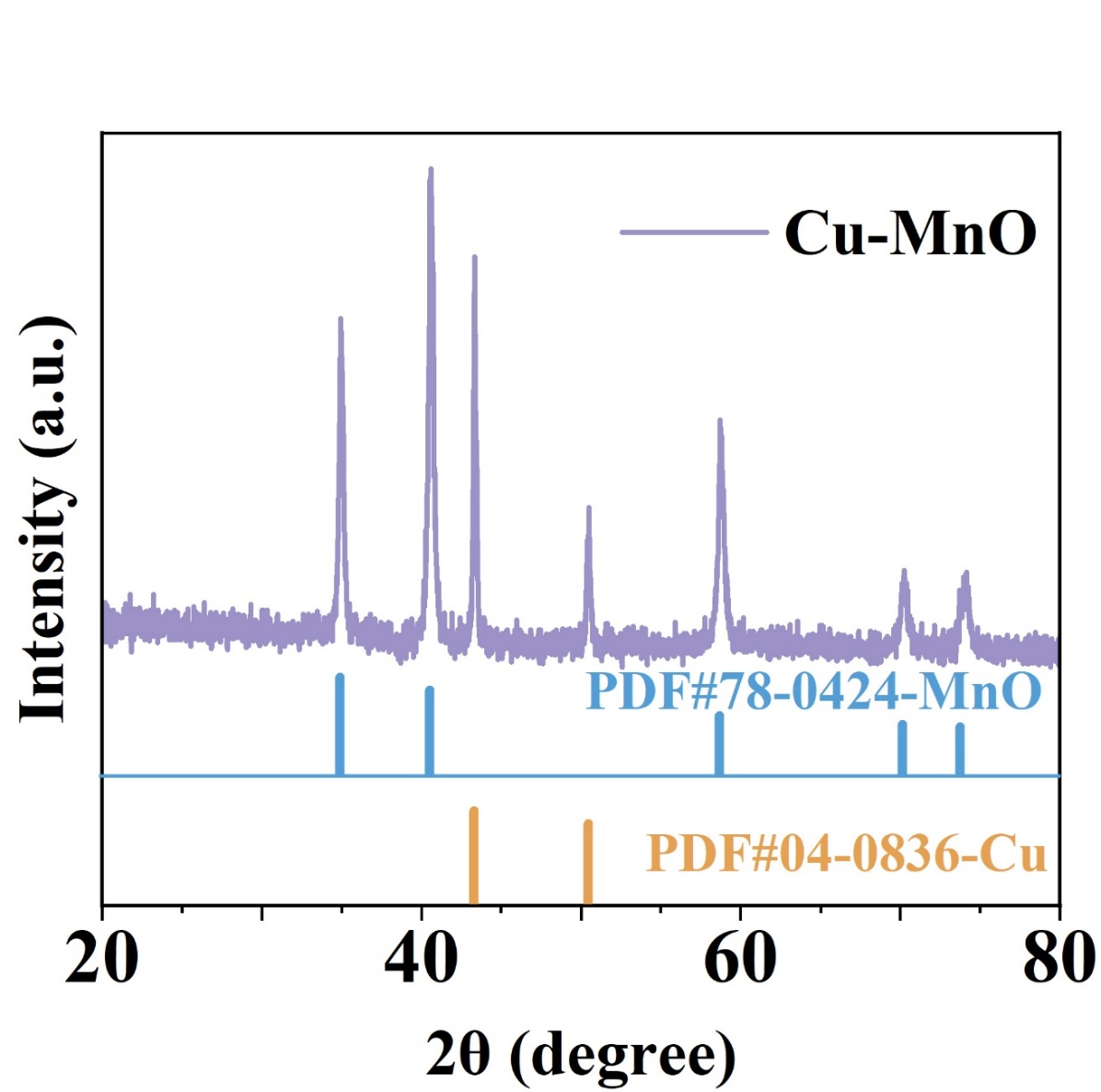


**Fig. S4.** XRD patterns of Cu-MnO.


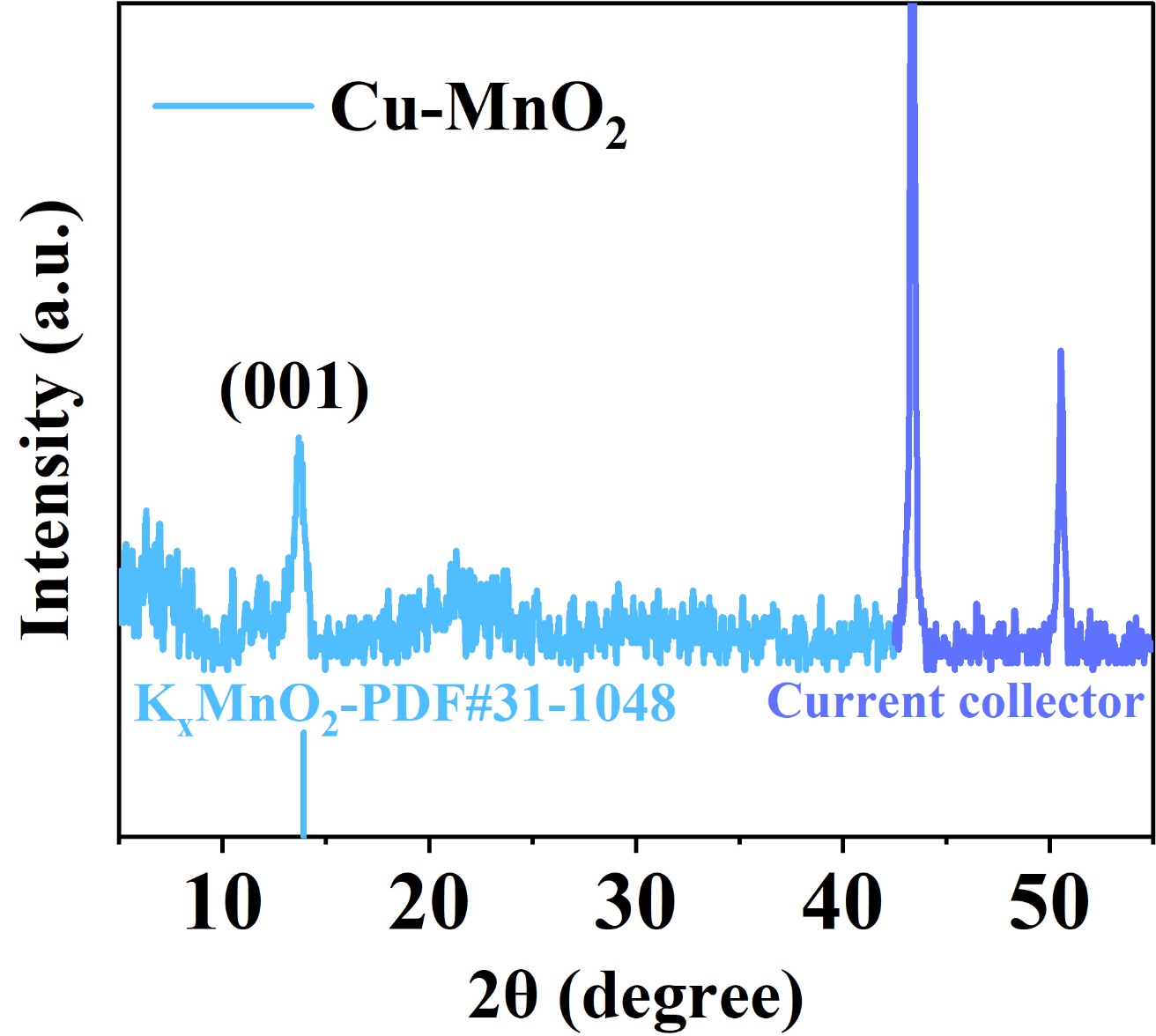


**Fig. S5.** XRD patterns of Cu-MnO_2_ at 1.9 V.

The XRD peak at 13.7° (Fig. 2b) corresponds to the (001) plane of K_x_MnO_2_.


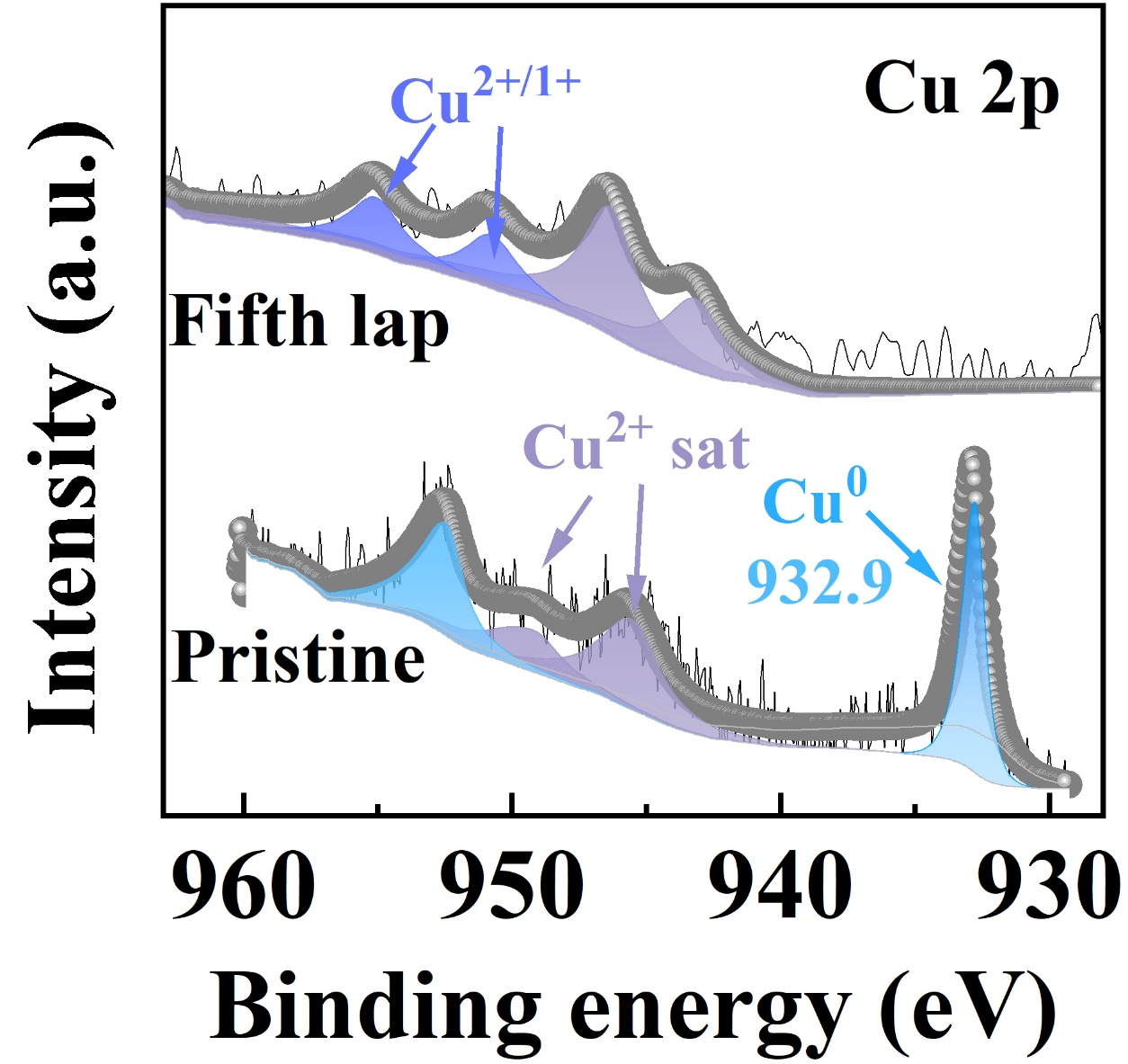


**Fig. S6**. Cu 2p of Cu-MnO_2_.

**
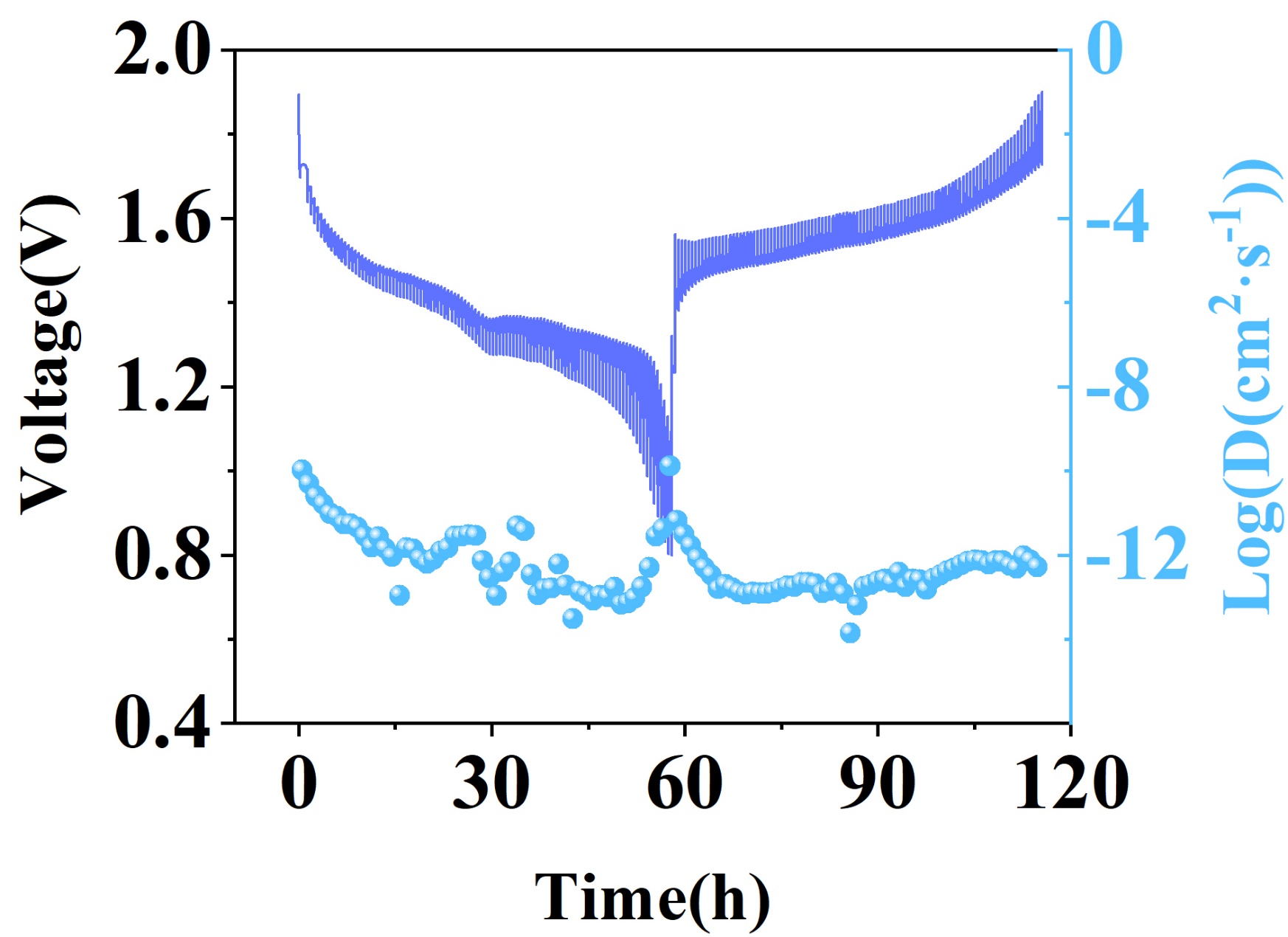
**

**Fig. S7**. GITT testing in ZMK electrolyte.

**
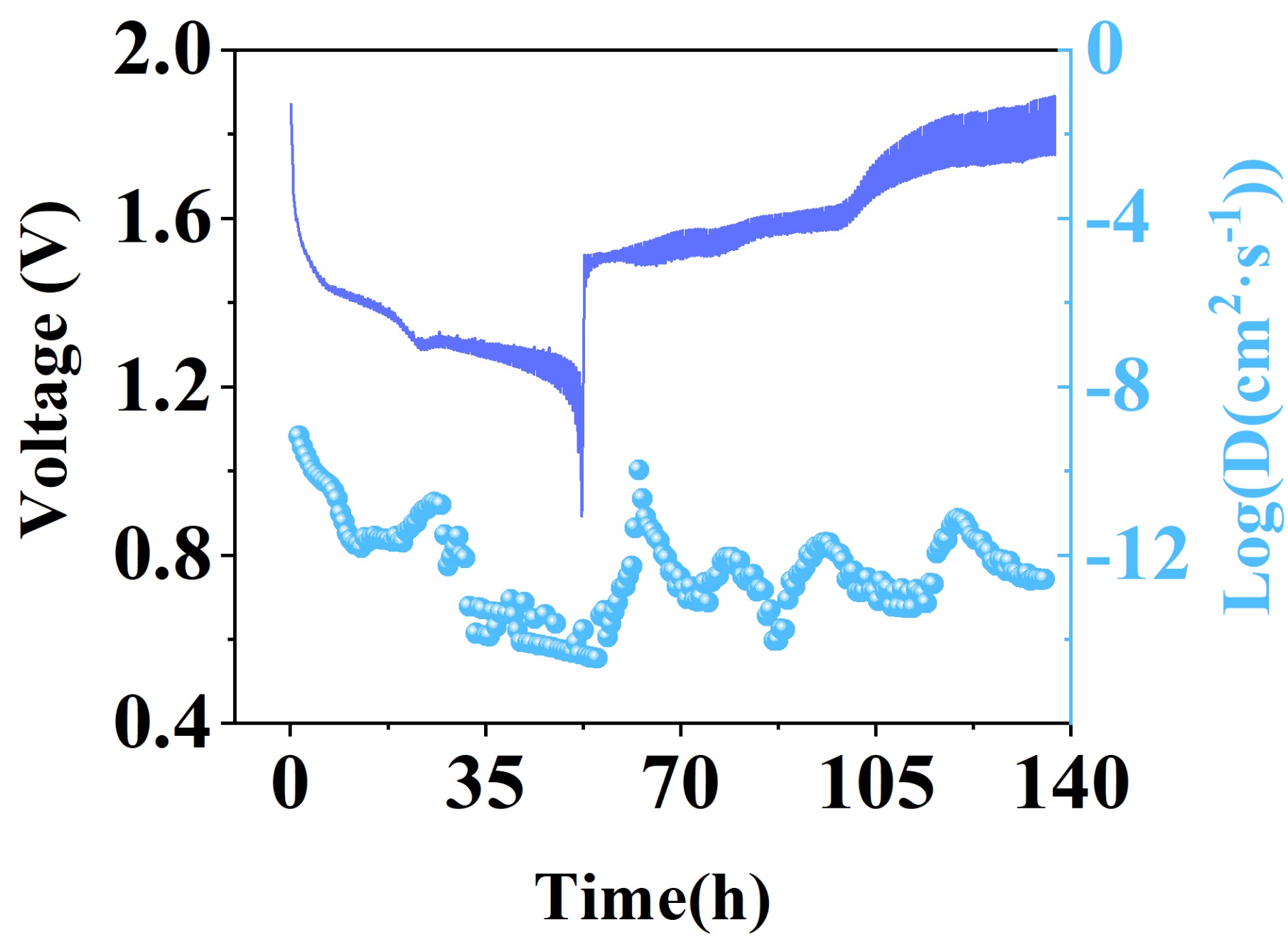
**

**Fig. S8**. GITT testing in ZM electrolyte.

**
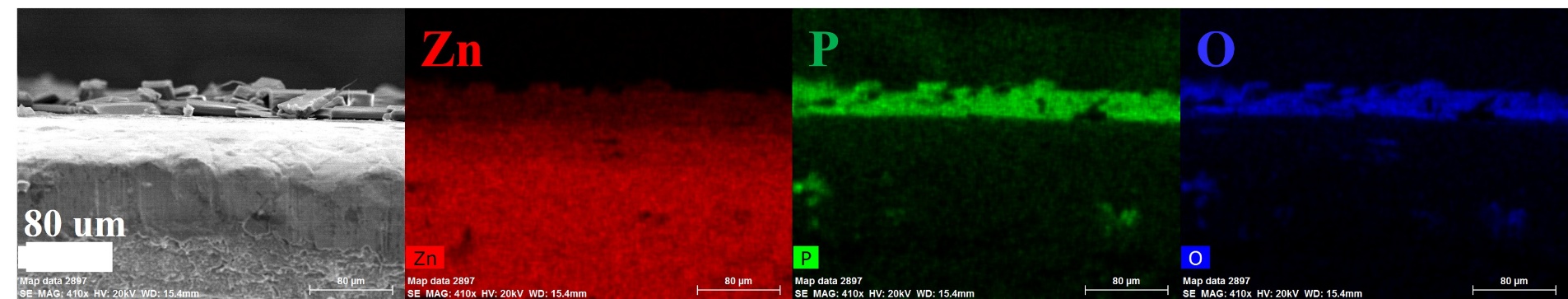
**

**Fig. S9**. Cross-sectional EDS image of Zn after 10 cycles in ZMK electrolyte.


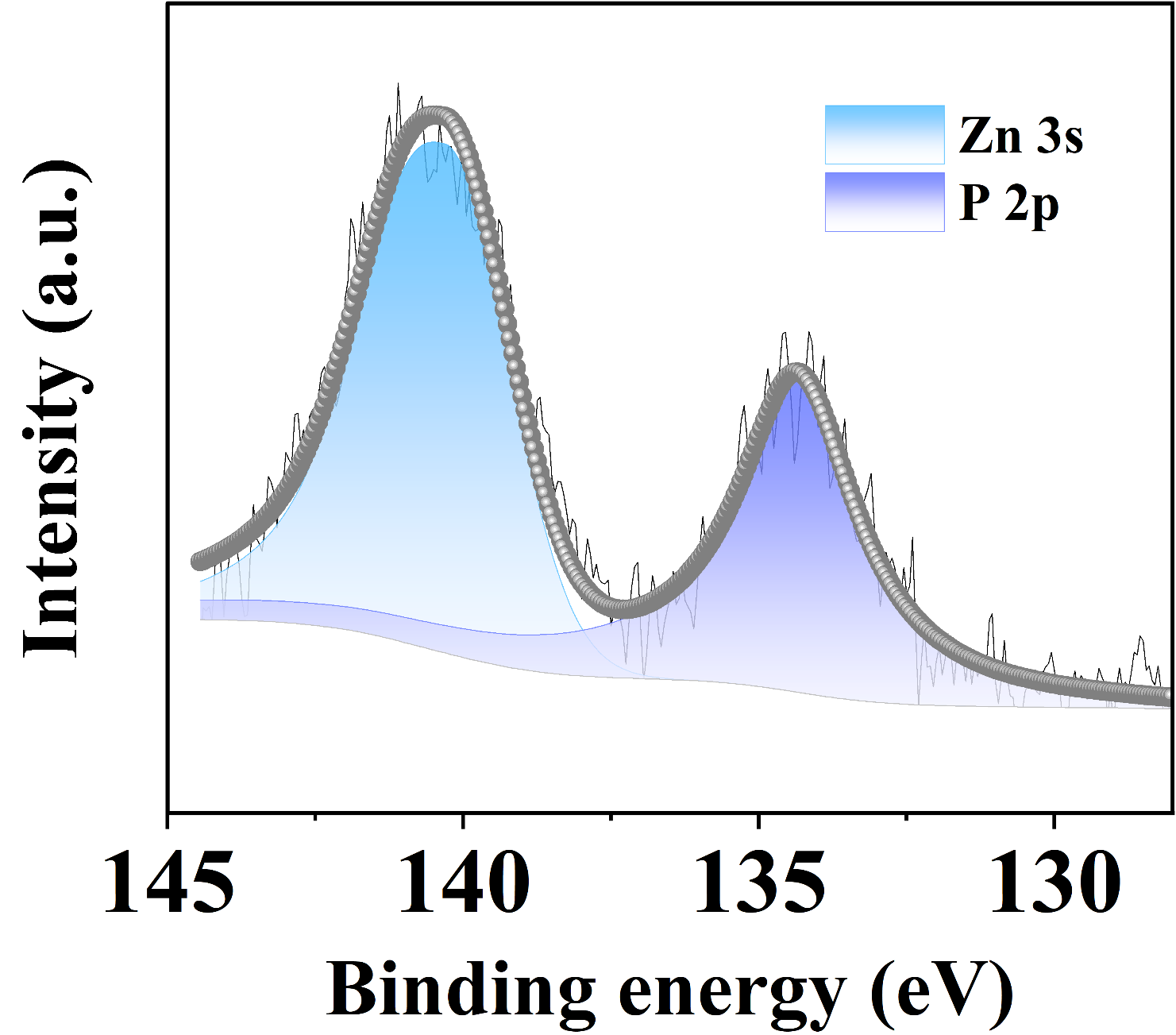


**Fig. S10**. P 2p spectra of zinc foil after 7 days of immersion in ZMK electrolytes.


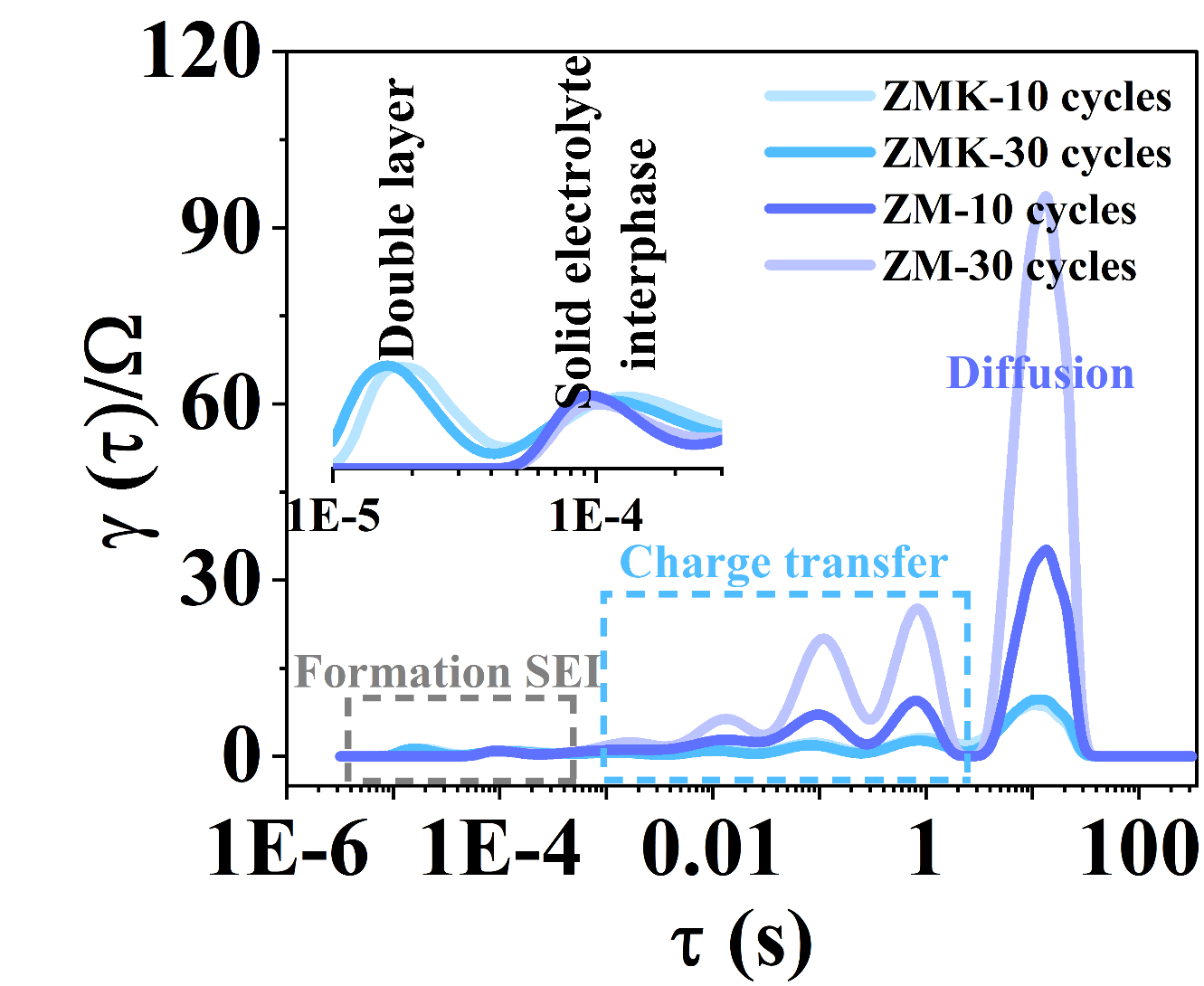


**Fig. S11**. DRT test of Zn//Zn symmetric battery in ZM/ZMK electrolyte with different circles.


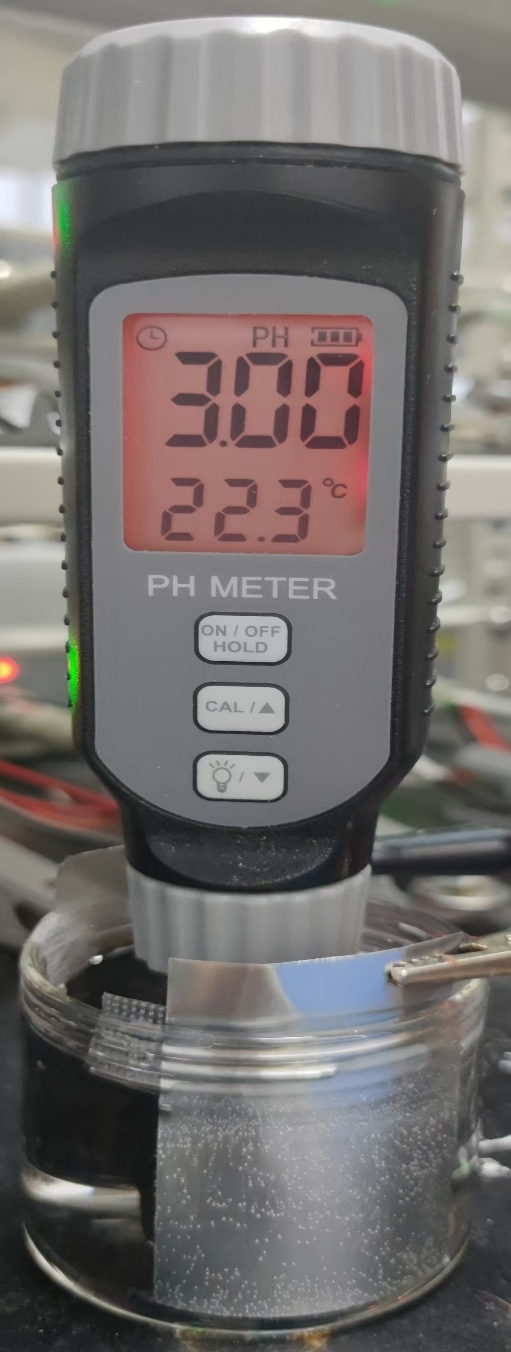


**Fig. S12**. pH testing apparatus diagram.

The pH meter used (model: PH818, brand: SMART) has a measurement range of 0.00-14.00, a resolution of 0.01 pH, and an accuracy of ±0.05 pH.


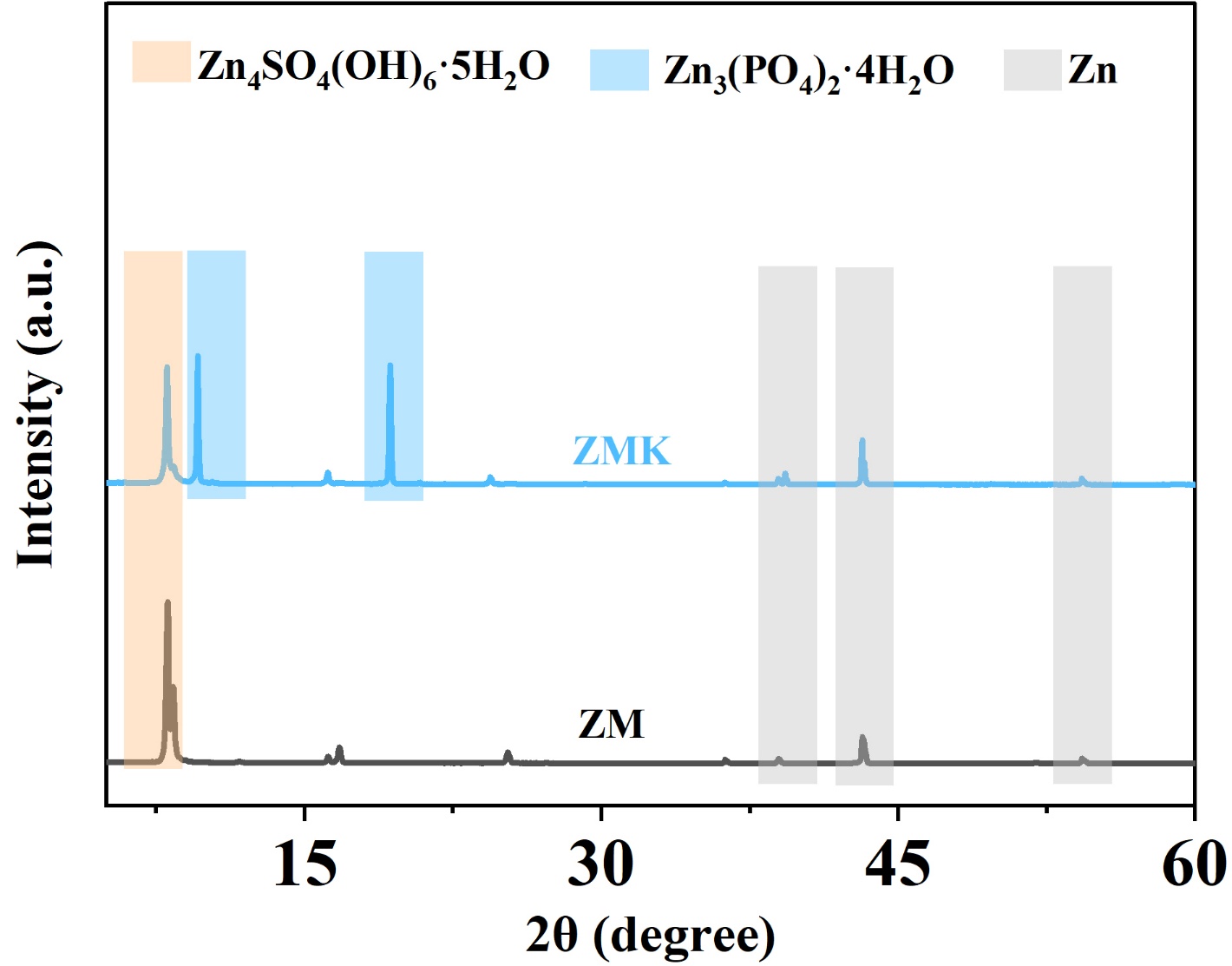


**Fig. S13**. XRD spectra of zinc foils after 7 days of immersion in ZM/ZMK electrolytes.


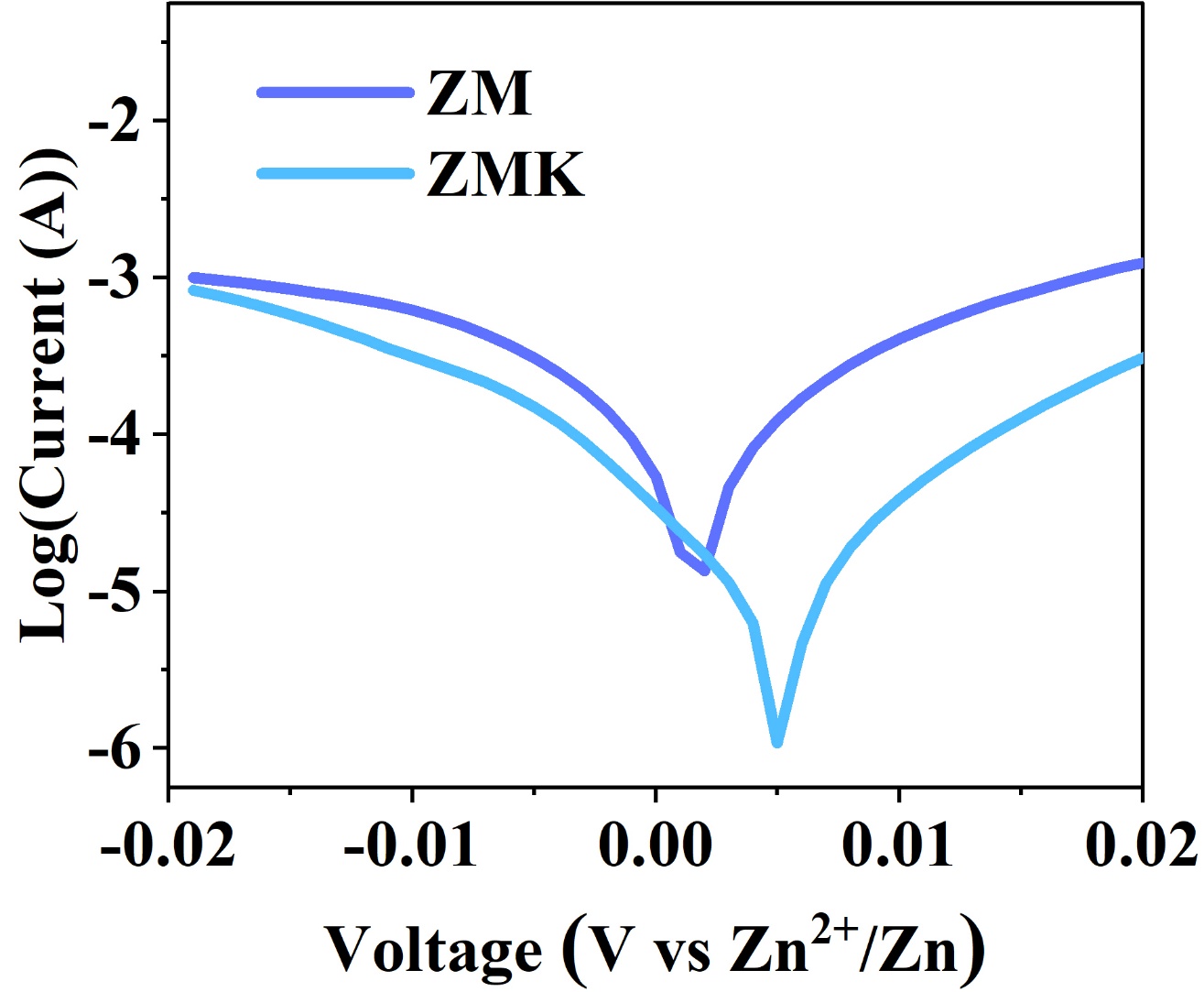


**Fig. S14**. Tafel plots of Zn//Zn cell in different electrolytes indicate the corrosion behavior.


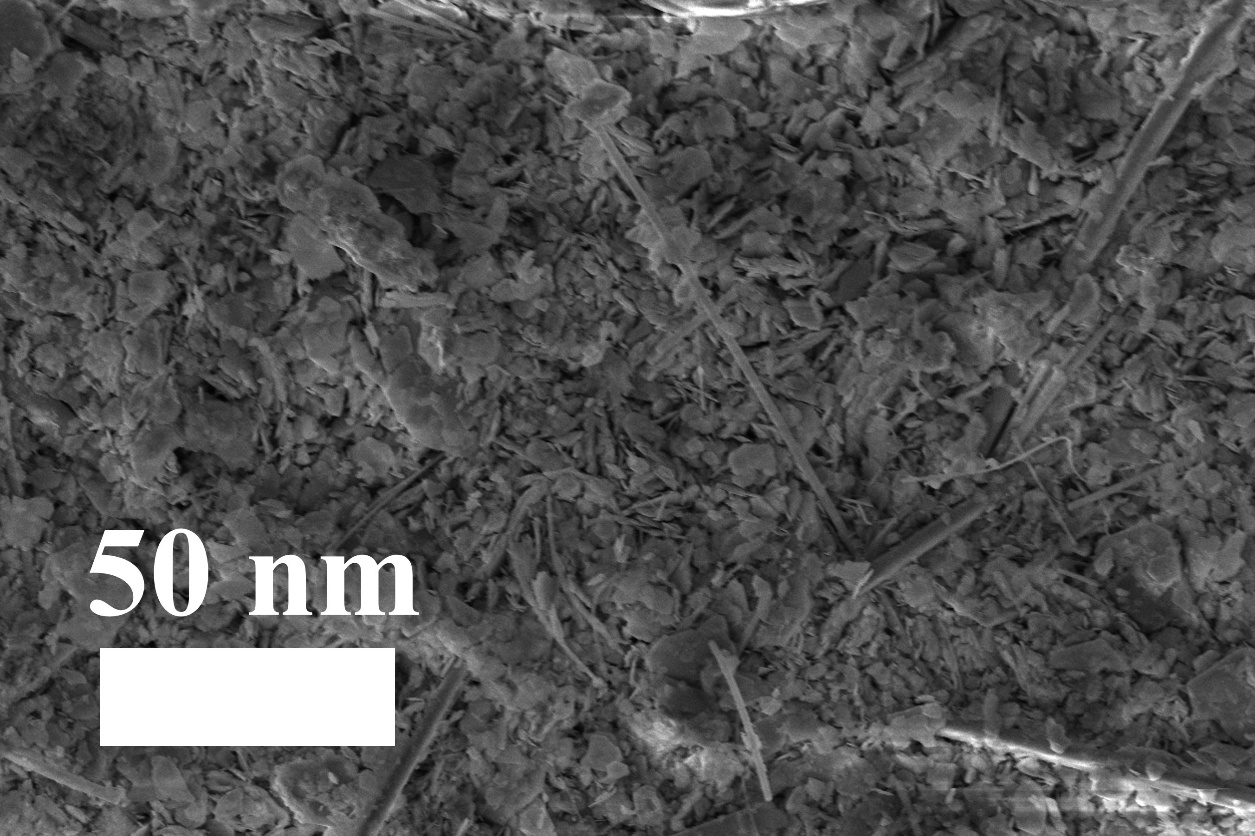


**Fig. S15**. SEM of the anode after 100000 cycles at ZMK electrolyte.


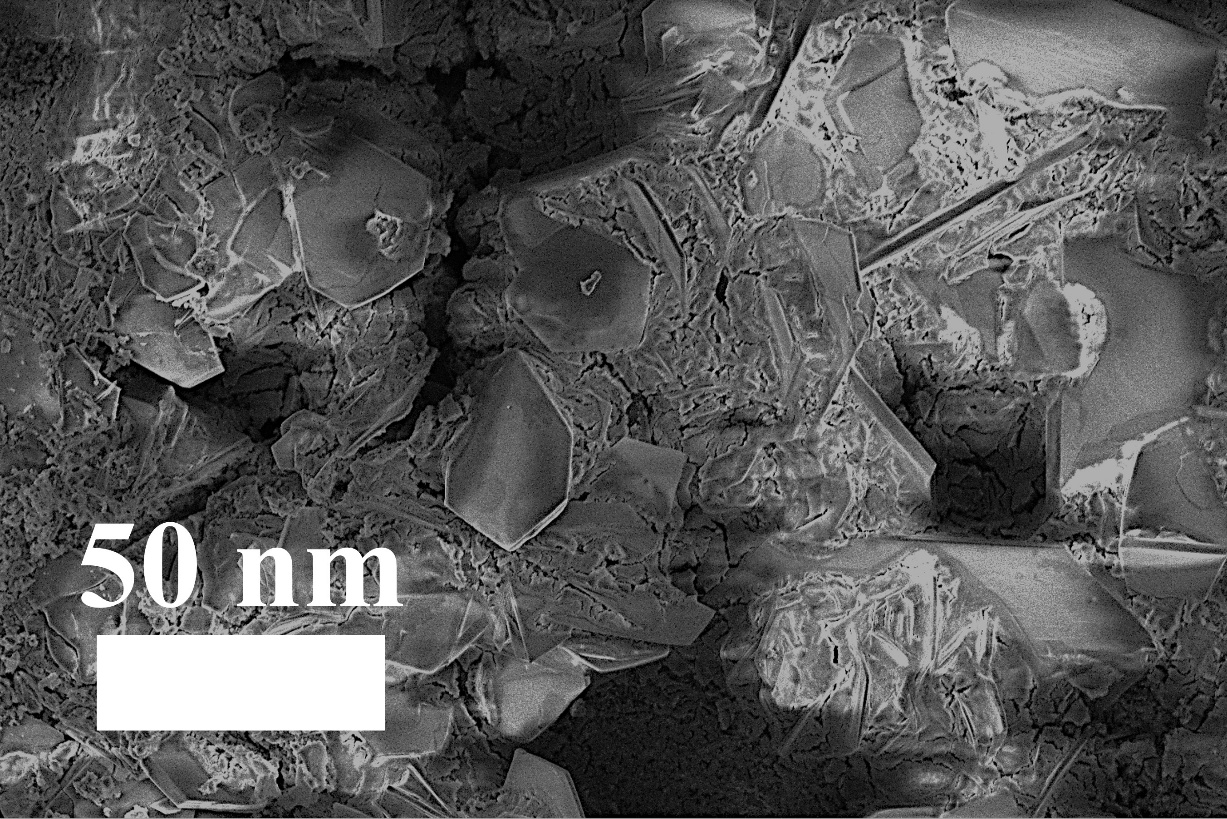


**Fig. S16**. SEM of the anode after cycles at ZM electrolyte.

**Table S1**. Comparison of the stability of HE-MnO/IMC with other manganese-based cathodes.

| Cathode Materials | Retention/Cycle/ Rate (A g^-1^) | | Rate Capability  (mAh g^-1^) | Ref |
| --- | --- | --- | --- | --- |
| Cu-MnO_2_ | | 77.3%/100000/5.0 | 274, 264, 211, 170, 76  (0.2, 0.5, 1.0, 2.0, 5.0) | This work |
| ZnCo-MnO/C | | 98.7%/3000/3.0 | 429, 269, 178, 128, 105, 81  (0.1, 0.5, 1.0, 1.5, 2.0, 3.0) | [S1] |
| MnO/MZ | | ~/11000/2.0 | 246, 111, 79, 67, 60, 55  (0.2, 0.5, 1, 2, 3, 4, 5) | [S2] |
| MnO@NGA | | 98.62%/2000/1.0 | 270, 252, 223, 169, 146, 100  (0.1, 0.2, 0.4, 0.8, 1.0, 2.0) | [S3] |
| Ni-MnO/PC | | 91.1%/6000/3.0 | 348, 330, 309, 290, 265, 202  (0.1, 0.2, 0.3, 0.4, 0.5, 1.0) | [S4] |
| MnS/MnO | | 174.2%/500/2.0 | 258, 252, 225, 189, 129  (0.1, 0.4, 0.8, 1.2, 2.0) | [S5] |
| KMg-MnO_2_ | | 94.0%/1000/0.5 | 398, 327, 278, 195, 100  (0.1, 0.3, 0.5, 1.0, 3.0) | [S6] |
| δ-MnO_2_ NDs | | 86.9%/5000/1.0 | 335, 327, 320, 274, 206, 125  (0.1, 0.2, 0.3, 0.5, 1.0, 2.0) | [S7] |
| BMO-6 | | 93%/10000 | 363, 332, 286, 251, 197, 103  (0.1, 0.2, 0.4, 0.6, 1.0, 3.0) | [S8] |
| ZMO QD@C | | 86.4%/1500/1.0 | 317, 269, 233, 204, 181, 159  (0.1, 0.2, 0.4, 0.6, 0.8, 1.0) | [S9] |
| Mn_3_O_4_/CNTs | | 84.1%/2800/2.0 | 421, 391, 311, 228, 139, 79  (0.1, 0.2, 0.5, 1.0, 2.0, 3.0) | [S10] |
| AMO | | 69%/10000/4.0 | 419, 304, 189, 105, 56  (0.5, 1.0, 2.0, 4.0, 8.0) | [S11] |
| Mg-MnO_2_ | | 50%/10000/1.5 | 385, 372, 298, 241, 219, 172  (0.3, 0.6, 1.5, 2.4, 3.0, 6.0) | [S12] |
| KMn_8_O_16_ | | 91%/2500/1.0 | 306, 283, 233, 180, 125, 106  (0.2, 0.5, 1.0, 2.0, 5.0, 10.0) | [S13] |
| S-MnO_2_ | | ~/1000/3.0 | 324, 311, 262, 205  (0.2, 0.5, 1.0, 2.0) | [S14] |
| Mg_0.9_Mn_3_O_7_·2.7H_2_O | | 92%/5000/5.0 | 312, 282, 245, 195, 164, 132  (0.2, 0.5, 1.0, 2.0, 3.0, 5.0) | [S15] |
| Cu-MnO_2_ | | ~ | 451, 405, 377, 296, 150  (0.1, 0.3, 0.5, 1.0, 3.0) | [S16] |
| C@PODA/MnO_2_ | | 89.3%/2000/2.0 | 310, 216, 179, 162, 122, 90  (0.1, 0.5, 1.0, 2.0, 5.0, 10.0) | [S17] |
| CuMn_2_O_4_ | | 83%/1800/3.0 | 262, 200, 173, 164, 120, 103  (0.2, 0.5, 0.8, 1.0, 3.0, 5.0) | [S18] |
| Vm-NiMn_3_O_7_ | | 91%/4000/5.0 | 254, 215, 173, 121  (0.5, 1.0, 2.0, 5.0) | [S19] |
| PANI | | 82%/10000/5.0 | 203, 186, 151, 128, 111, 94  (0.1, 0.2, 0.5, 1.0, 2.0, 5.0) | [S20] |
| Cu-HHTP/MXene | | 92.9%/1000/4.0 | 251, 245, 211, 196, 171  (0.1, 0.2, 0.5, 1.0, 4.0) | [S21] |
| V_2_O_5_ | | 82.2%/4000/5.0 | ~ | [S22] |
| O_v_-ZVO | | 83%/2000/2.0 | 402, 365, 345, 295, 250  (0.1, 0.2, 0.5, 1.0, 2.0) | [S23] |

**SReferences**

[S1] K. Sun, Y. Shen, J. Min, J. Pang, Y. Zheng, T. Gu, G. Wang, L. Chen, *Chem. Eng. J.* **2023**, 454, 140394.

[S2] Y. Liu, Z. Qin, X. Yang, X. Sun, *Adv. Funct. Mater.* **2022**, 32, 2106994, 2106994.

[S3] W. Zheng, G. Liang, Q. Liu, J. Li, J. Yuwono, S. Zhang, V. Peterson, Z. Guo, *Joule* **2023**, 7, 2732.

[S4] Y. Y. Chen, X. S. Hu, X. D. Chen, J. H. Liu, Y. Huang, D. P. Cao, *Chem. Eng. J.* **2023**, 478, 147411, 147411.

[S5] F. Tang, X. Wu, Y. Shen, Y. Xiang, X. Wu, L. Xiong, X. Wu, *Energy Storage Mater.* **2022**, 52, 180.

[S6] F. Jing, Y. Liu, Y. Shang, C. Lv, L. Xu, J. Pei, J. Liu, G. Chen, C. Yan, *Energy Storage Mater.* **2022**, 49, 164.

[S7] H. Tang, W. Chen, N. Li, Z. Hu, L. Xiao, Y. Xie, L. Xi, L. Ni, Y. Zhu, *Energy Storage Mater.* **2022**, 48, 335.

[S8] Y. Ma, M. Xu, R. Liu, H. Xiao, Y. Liu, X. Wang, Y. Huang, G. Yuan, *Energy Storage Mater.* **2022**, 48, 212.

[S9] S. Deng, Z. Tie, F. Yue, H. Cao, M. Yao, Z. Niu, *Angew. Chem. Int. Ed.* **2022**, 61, e202115877.

[S10] X. Guo, H. Sun, C. Li, S. Zhang, Z. Li, X. Hou, X. Chen, J. Liu, Z. Shi, S. Feng, *J. Energy Chem.* **2022**, 68, 538.

[S11] H. Yao, H. Yu, Y. Zheng, N. Li, S. Li, D. Luan, X. Lou, L. Yu, *Angew. Chem. Int. Ed.* **2023**, 62, e202315257.

[S12] J. Xia, Y. Zhou, J. Zhang, T. Lu, W. Gong, D. Zhang, X. Wang, J. Di, *Small* **2023**, 19, 2301906.

[S13] G. Cui, Y. Zeng, J. Wu, Y. Guo, X. Gu, X. Lou, *Adv. Sci.* **2022**, 9, 2106067, 2106067.

[S14] Y. Zhao, P. Zhang, J. Liang, X. Xia, L. Ren, L. Song, W. Liu, X. Sun, *Energy Storage Mater.* **2022**, 47, 424.

[S15] J. Li, N. Luo, L. Kang, F. Zhao, Y. Jiao, T. J. Macdonald, M. Wang, I. P. Parkin, P. R. Shearing, D. J. L. Brett, G. Chai, G. He, *Adv. Energy Mater.* **2022**, 12, 2201840, 2201840.

[S16] J. Zhang, W. Li, J. Wang, X. Pu, G. Zhang, S. Wang, N. Wang, X. Li, *Angew. Chem. Int. Ed.* **2023**, 62, e202215654.

[S17] Y. Zhao, R. Zhou, Z. Song, X. Zhang, T. Zhang, A. Zhou, F. Wu, R. Chen, L. Li, *Angew. Chem. Int. Ed.* **2022**, 61, e202212231.

[S18] G. Yang, K. Ma, C. Wang, *Adv. Energy Mater.* **2024**, 14, 2303695.

[S19] F. Zhao, J. Li, A. Chutia, L. Liu, L. Kang, F. L. Lai, H. Dong, X. Gao, Y. Tan, T. Liu, I. P. Parkin, G. He, *Energy Environ. Sci.* **2024**, 17, 1497.

[S20] H. Li, Y. Liu, L. Huang, J. Xin, T. Zhang, P. Liu, L. Chen, W. Guo, T. Gu, G. Wang, *J. Mater. Chem. A* **2023**, 11, 5179.

[S21] Y. Wang, J. Song, W. Wong, *Angew. Chem. Int. Ed.* **2023**, 62, e202218343.

[S22] S. Liu, J. Vongsvivut, Y. Wang, R. Zhang, F. Yang, S. Zhang, K. Davey, J. Mao, Z. Guo, *Angew. Chem. Int. Ed.* **2023**, 62, e202215600.

[S23] J. Ye, P. Li, H. Zhang, Z. Song, T. Fan, W. Zhang, J. Tian, T. Huang, Y. Qian, Z. Hou, N. Shpigel, L. Chen, S. Dou, *Adv. Funct. Mater.* **2023**, 33, 2305659, 2305659.
